# Design of selective COX-2 inhibitors in the (aza)indazole series. Chemistry, *in vitro* studies, radiochemistry and evaluations in rats of a [^18^F] PET tracer

**DOI:** 10.1080/14756366.2018.1501043

**Published:** 2018-10-26

**Authors:** Jonathan Elie, Johnny Vercouillie, Nicolas Arlicot, Lucas Lemaire, Rudy Bidault, Sylvie Bodard, Christel Hosselet, Jean-Bernard Deloye, Sylvie Chalon, Patrick Emond, Denis Guilloteau, Frédéric Buron, Sylvain Routier

**Affiliations:** a ICOA, UMR CNRS 7311, University of Orleans, Orleans, France;; b UMR 1253, iBrain , Université de Tours, Inserm, Tours, France;; c CERRP, Centre d’Etude et de Recherche sur les Radiopharmaceutiques, Tours, France;; d CHRU, de Tours, Tours, France;; e INSERM CIC 1415, University of François-Rabelais de Tours, Tours, France;; f Biopôle Clermont-Limagne, Laboratoires Cyclopharma, Saint-Beauzire, France

**Keywords:** Cyclooxygenase, neuroinflammation, NSAID, radiolabeling, PET, boronic ester, (aza)indazoles

## Abstract

A series of novel derivatives exhibiting high affinity and selectivity towards the COX-2 enzyme in the (aza) indazole series was developed. A short synthetic route involving a bromination/arylation sequence under microwave irradiation and direct C–H activation were established in the indazole and azaindazole series respectively. *In vitro* assays were conducted and structural modifications were carried out on these scaffolds to furnish compound **16** which exhibited effective COX-2 inhibitory activity, with IC_50_ values of 0.409 µM and an excellent selectivity versus COX-1. Radiolabeling of this most potent derivative [^18^F]**16** was achieved after boron ester release and the tracer was evaluated *in vivo* in a rat model of neuroinflammation. All chemistry, radiochemistry and biological experimental data are discussed.

## Graphical Abstract

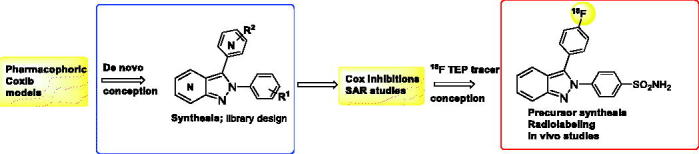

## Introduction

An inflammatory reaction is a ubiquitous effective protective mechanism, which includes the cascade activation of coordinated chemical and cellular events. The role of inflammation is to restore tissue homeostasis^1^. However, it can have either a beneficial effect when it promotes repair or adverse consequences when it is excessive or long-lasting. Compared to other organs, the brain is characterised by a low regenerative capacity and specific immune processes due to the presence of the blood-brain-barrier (BBB). The immune response of the brain to various injuries is called neuroinflammation, and includes a number of events, the main one being the activation of microglial cells^2^. During this process, microglia change from a resting to an activated state, which can either mediate protective and regenerating mechanisms or on the contrary aggravate injury, contributing to neurodegeneration^3^. Neuroinflammation is involved in a number of neurodegenerative disorders such as Alzheimer’s disease (AD) and Parkinson’s disease (PD) as well as in several neuropsychiatric disorders such as autism, schizophrenia, and depression^4–6^. The *in vivo* detection and quantification of neuroinflammation in several brain diseases can therefore, be of high value for a better understanding of pathophysiological mechanisms, early diagnosis, and identification of new therapeutic approaches. In this context, a large panel of molecular targets can be envisaged for positron emission tomography (PET) exploration^7^.

Among all the molecular pathways involved in the inflammation process, the cyclooxygenase (COX) enzyme that contributes to the subsequent production of prostaglandins clearly plays a central role. COX-2 is an inducible enzyme, which is expressed at high concentrations at inflammation sites and malignant transformations compared to most normal tissues. This, associated with the availability of COX-2-selective inhibitors, makes this enzyme an ideal target in order to image inflammation^8^^,^^9^.

**Figure 1.**
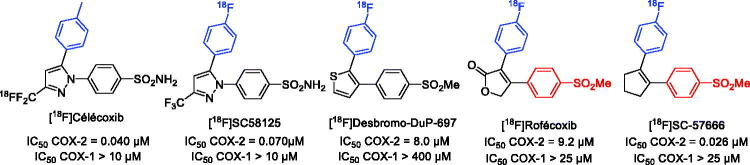
Structure of some [^18^F]coxib tracers.

Several selective COX-2 inhibitors have been reported in the literature, among them, the Coxib family (celecoxib, rofecoxib), a non-steroidal anti-inflammatory drug class (NSAIDs) which has been extensively studied^10^. Several [^18^F]PET radiotracers to image COX-2 have been developed over the past decade (Figure 1)^8^^,^^9^. Although some of them have proved useful to explore inflammation in a pre-clinical colorectal cancer model, no specific COX-2 radiotracers are available to visualise brain inflammation because of a poor brain penetrance which remains the main challenge for all PET agents targeting the central nervous system (CNS)^11^^,^^12^.

**Figure 2.**
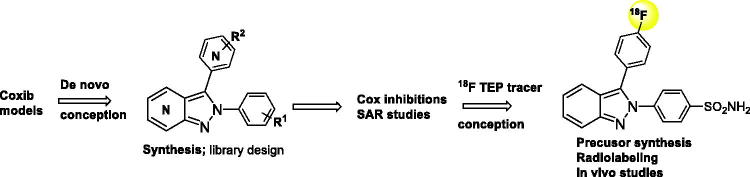
Objectives of the study.

The present study, therefore aimed to develop a novel series of selective COX-2 inhibitors using novel 2,3-di(het)arylated (aza)indazole series derivatives and to establish structure-activity relationships (Figure 2). After developing the synthetic pathways and proving their efficiency in a library-building strategy, each new compound was evaluated for its activity on COX-2 as well as its selectivity over COX-1 when relevant. The most potent derivative compound was converted to 18 F PET tracer to perform *in vivo* studies. Each step of this study, aimed at the *de novo* conception of potent ^18^ F radiolabeled ligands targeting COX-2, is presented and the results discussed.

## Results and discussion

### Chemistry

**Scheme 1.**
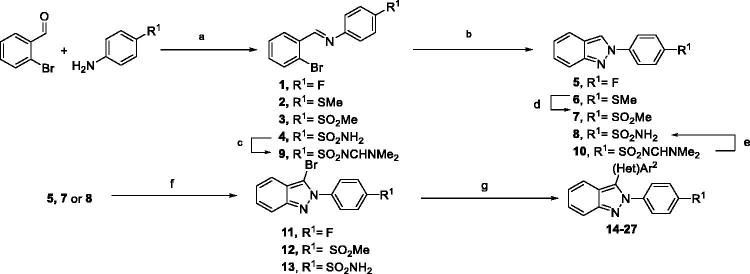
Synthesis of di(het)arylated indazoles **14–27**. Reagents and conditions: (a) 2- bromobenzaldehyde (1.0 equiv), aniline (1.01 equiv), MgSO_4_ (5.0 equiv), THF, 100 °C, μW, **1**: 10 min, 99%; **2**: 15 min, 99%; **3**: 1.5 h, 95%; **4**: 1.5 h, 90%; (b) NaN_3_ (2.5 equiv), CuI (0.1 equiv), DMSO, 150 °C, μW, 5 min, **5**: 99%; **6**: 99%; **7**: 17%; **10**: quant TLC; (c) DMF.DMA (2.0 equiv), THF, r.t., 1 h, 98%; (d) Oxone (2.1 equiv), MeOH/H_2_O (2/1), r.t., 20 h, 83%; (e) NaOH (3.0 equiv), 150 °C, μW, 5 min then HCl conc., 85% from **9**; (f) Br_2_ (1.0 equiv), AcOH/MeOH/CH_2_Cl_2_ (2/1/0.1), r.t., 4 h, **11**: 95%; **12**: 99%; **13**: 92%; (g) (Het)Ar^2^-B(OH)_2_ (1.2 equiv.), Cs_2_CO_3_ (3.0 equiv), Pd(PPh_3_)_4_ (0.1 equiv), dioxane, 150 °C, μW, 1 h, structures and yields are indicated in Table 2.

The *2H*-indazoles **5**–**8** were synthesised using a two-step approach including a Schiff base formation followed by intramolecular cyclisation^13^^,^^14^ using microwave irradiation to reduce the reaction time of each step from 12 h to a few minutes. The condensation of aniline with 2-bromobenzaldehyde in presence of MgSO_4_ under microwave activation allowed access to imine **1**–**4** in excellent yields. *N-2* arylated-indazoles were obtained using a copper-catalysed reaction with sodium azide, and compounds **5** and **6** were isolated in 95% and 56% yields, respectively (Scheme 1). However, no reaction occurred in the presence of methanesulfonyl **3** or methanesulfonamide **4**. This lack of reactivity prompted us to oxidise the thiomethyl function of **6** under classical conditions to sulfone **7** which was isolated in 83% yield. Concerning the access to **8**, the presence of the sulfonamide acidic function on **4** seems to inhibit the intramolecular reaction. To counteract this effect, we decided to use *N*, *N*-dimethylformamidine as the sulfonamide protecting group. After synthesising **9** from **4** using DMF.DMA as a reagent, the near quantitative annelation furnished **10** (TLC) which was immediately subjected to deprotection in basic media and led successfully to the desired indazole **8** in an 85% overall yield.

The bromination step occurred smoothly and the desired products **11**–**13** were isolated in excellent yields. For the next *C-*3 (het)arylations, we successfully achieved the Suzuki-Miyaura cross-coupling reactions using several commercially available arylboronic acids (1.2 equiv.) under classical conditions^15–17^. The desired products **14**–**27** were isolated in moderate to excellent yields (Table 2). Most of the reactions appeared to be complete during the monitoring and variations in the efficiency of the cross-coupling reaction were mostly related to the purification step. Details of the structures and yield in final products are given in Table 2.

**Table 1.** Synthesis of 2-aryl azaindazoles **36–38**. 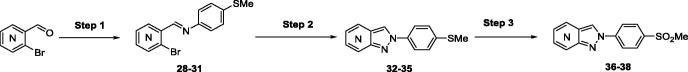

Entry	Step 1		Step 2		Step 3	
	Product (Yield %)^[a]^^,^^[b]^		Product (Yield %)^[a]^		Product (Yield %)^[a]^	
1		**28** (99)		**32** (29)		**36** (66)
2		**29** (62)		**33** (ND)		
3		**30** (99)		**34** (45)		**37** (34)
4		**31** (99)		**35** (23)		**38** (58)

[a] Yields are indicated in isolated products

[b] Irradiation time 15 min. ND: Not Detected. Step 1: (a) 2- bromobenzaldehyde (1.0 equiv), aniline (1.01 equiv), MgSO_4_ (5.0 equiv), THF, 100 °C, μW, 15 min. Step 2: NaN_3_ (2.5 equiv), CuI (0.1 equiv), DMSO, 150 °C, μW, 5 min. Step 3: Oxone (2.1 equiv), MeOH/H_2_O (2/1), r.t., 20 h.

**Table 2.** Cox-2 inhibition.

Entry	Product (Yield %)^[b]^		IC_50_ COX-2 (µM)^[a]^	Entry	Product (Yield %)^[b]^		IC_50_ COX-2 (µM)^[a]^
1		**14** (95)	1.459 ± 0.5	11		**23** (24)	>25
2		**15** (47)	2.296 ± 0.5	12		**24** (85)	2.977 ± 0.4
3		**39** (63)	>25	13		**25** (46)	>25
4		**40** (75)	4.421 ± 0.6	14		**26** (94)	4.461 ± 0.4
5		**41** (79)	4.750 ± 0.6	15		**27** (85)	>25
6		**18** (95)	1.028 ± 0.2	16		**16** (73)	**0.409**±0.3
7		**19** (98)	>25	17		**17** (59)	4.200 ± 0.4
8		**20** (72)	1. 923 ± 0.4	18		DuP-697	0.039 ± 0.024
9		**21** (91)	1.100 ± 0.3	19		Celecoxib	0.076 ± 0.054
10		**22** (85)	>25				

[a] Values are means of triplicates excepted for DuP-697 (*n* = 27) and Celecoxib (*n* = 5) which serves as reference in the assays

[b] Yields are thus obtained at the final step reported in Scheme 1.

To be able to define SAR, we next studied the incorporation of a nitrogen atom in the central heterocyclic scaffold and a switch to the azaindazole series. To obtain the expected derivatives, we adapted the previous synthetic strategy and employed, as starting materials, pyridines that were brominated and formylated in vicinal positions. While the first synthetic pathway reaction afforded imines in near quantitative yields, building the azaindazoles and the following sulfur oxidation steps appeared hazardous but fortunately afforded **36–38** as pure compounds (Table 1).

**Scheme 2.**
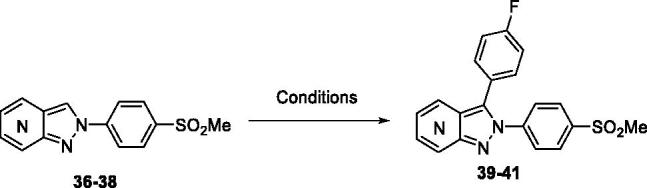
Synthesis of fluorinated azaindazoles. Reagents and conditions: 4-Iodofluorobenzene(1.1 equiv), Ag_2_CO_3_ (1.0 equiv), Pd(dppf)Cl_2_.CH_2_Cl_2_ (0.05 equiv), PPh_3_ (0.10 equiv), H_2_O, 50 °C, 18 h, **39**: 4-aza 63%; **40**: 6-aza 75%; **41**: 7-aza 79%.

The last step consisted in introducing an aryl moiety in *C*-3 position. In the literature, the sole methodology to describe this functionalization in the azaindazole series used a direct arylation strategy^15–17^. In the present study, the reaction required employing a catalytic system including Pd(dppf)Cl_2_.CH_2_Cl_2_, Ag_2_CO_3_ as base and water as solvent. This powerful system was used starting from compounds **36**–**38** and led to 2,3-diarylazaindazole derivatives **39–41** in fairly good yields (Scheme 2).

### *In vitro* enzymatic assays

The derivatives **14–27** and compounds **39–41** were evaluated using a COX Inhibitor Screening Kit from Cayman Chemical Company (Table 2) to measure COX activity. Our set of molecules, designed for COX-2 inhibition, appeared to be very selective towards COX-1 as no efficient inhibition of this enzyme was detected at 30 µM for any of the derivatives tested. In the indazole series, all the derivatives showed an activity against the COX-2 enzyme. The SAR study showed that the best position for the 4-sulfonylaryl moiety remained the *N*-2 position since its shift to *C*-3 led to a decrease in COX-2 activity (entries 1–2). Compound **14** showed an interesting IC_50_ around 1 µM whereas its isomer analog **15** was active with an IC_50_ up to 2 µM. Despite our synthetic effort, the azaindazole derivatives **41** and **42** were 4-fold less efficient than their aromatic analog **14** (entries 3–4). The position of nitrogen is related to the efficiency as only 4- or 5- azaindazoles **40** and **41** versus **39** exhibited an IC_50_ value against COX-2 at an acceptable level.

In the indazole series, the next modulations concerned the *C*-3 aromatic moiety. Its bi-substitution by methyl and fluorine (in *C*-4 and *C*-3 respectively) led to the most active derivative **18** (entry 6). In *C*-4, an ethyl ether group considerably diminished the activity (entry 8), which was partially restored when fluorine was replaced by a CF_3_ group. To prove that the oxygen electron donating effect is involved in the activity loss, we replaced the ether residue by a chlorine atom and observed that **21** (entry 9) was as active as **18**. In order to modulate the electronic density in *C*-9, we replaced the *C*-3 aryl group with a heterocyclic scaffold. 2 or 2,6-fluoropyridines and 3-methoxy groups led only to inactive derivatives (entries 10,12) whereas the 2-alkoxy pyridines **24** and **26** exhibited moderate activity (entries 10–12 *vs* 13–14). Finally pyrimidine does not appear suitable to improve the pharmacophoric model as **27** (entry 15) was totally inactive. In conclusion, these modulations indicated that each modulation in the *C*-3 indazolic position needs to be finely tuned to conserve activity on the COX-2 target, which is very sensitive to electronic, lipophilicity and steric parameters.

To complete our study, we replaced the methylsulfonyl group with a sulfonamide group. The analog of **14** afforded the most active derivative **16,** which showed a very interesting IC_50_ at 400 nM. Again, modification of the *C*-3 aryl functional group position led to a strong variability in efficiency as the fluorine displacement from the para to the meta position (**17**, entry 17) led to a 10-fold decrease in activity.

### Radiolabeling studies

**Scheme 3.**
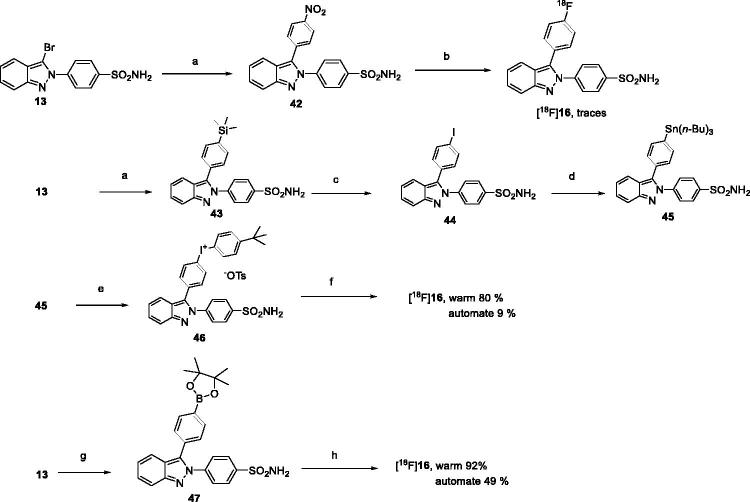
Methods for radiolabeling. Reagents and conditions: (a) corresponding boronic acid (1.2 equiv), Cs_2_CO_3_ (3.0 equiv), Pd(PPh_3_)_4_ (0.1 equiv), dioxane, 150 °C, μW, 1 h, **42** 53%, **43** 69%; (b) [K/K_222_]^+^^18^F^–^, DMF, 130 °C, μW, 20 min; (c) ICl (2.0 equiv), CH_2_Cl_2_, 0 °C then t.a., 1.5 h, 99%; (d) *n-*Bu_6_Sn_2_ (3.3 equiv), Pd(PPh_3_)_4_ (0.1 equiv), dioxane, 90 °C, 2 h, 33%; (e) Moser reagent (1.5 equiv), CH_3_CN/CH_2_Cl_2_ (1/1), r.t., 18 h, 73%; (f) [K/K_222_]^+^ 18F^–^, DMF, Cu(OTf)_2_ cat, 100 °C, μW, 10 min; (g) 1,4-bis(4,4,5,5-tetramethyl-1,3,2-dioxaborolan-2-yl)benzene (3.0 equiv), Na_2_CO_3_ (6.5 equiv.), Pd(PPh_3_)_4_ (0.02 equiv), dioxane, 100 °C, μW, 40 min, 52%; (h) KOTf; K_2_CO_3_, [^18^F]KF, Cu(OTf)_2_ cat., DMF, pyridine, 130 °C, 20 min.

From our *in vitro* experiments, the sulfonylamide derivative **16** was found to be the most selective COX-2 inhibitor in the *de novo* built (aza)indazole library. This activity, which is the direct reflection of the specific biological target affinity, indicated that it could be a potent ^18^ F probe candidate. Three different labeling strategies were envisaged to obtain the desired radiotracer (Scheme 3). As the easiest way of precursor preparation, we first tried starting from the nitro derivative **42,** which was prepared from **13** after a Suzuki-Miyaura cross-coupling reaction in a 53% yield. Under thermal conditions direct nucleophilic substitution always failed. In fact, traces of [^18^F]**16** were only detected under microwave irradiation (2–3%). Then we switched to the use of a more complex iodonium salt derivative **46,** which was obtained after 4 steps from **13**. First a Suzuki-Miyaura cross-coupling reaction led to **43** in a 69% yield. Electrophilic iodination with ICl afforded^18^, in a near quantitative yield, the 4-iodo phenyl derivative **44**, which was next transformed into tri *n*-butyl stannyl analog **45** under palladium catalysis in a moderate yield. Finally, Koser reagent smoothly afforded the attempted iodonium salt **46** in a satisfying manner. Based on the literature^19^^,^^20^, the radiolabeling reaction was performed in DMF using Cu(OTf)_2_ as catalyst under microwave irradiation at 100 °C. In warm conditions the incorporation of 18 F, up to 60%, occurred but surprisingly in hot conditions with automation, the pure formulated derivative [^18^F]**16** was obtained with a very poor yield of 9%. So, a third strategy requiring the use of boronic ester as leaving group was conducted. Starting from **13**, the mono Suzuki reaction led to boronic ester **46** in a 52% yield.

Based on recent work^21^^,^^22^, the nucleophilic substitution was achieved using an eluent solution containing the 18 F from QMA and KOTf and K_2_CO_3_ in CH_3_CN/water and pyridine. This solution was added to a solution which contained precursor **47,** DMF as solvent and Cu(OTf)_2_ as a catalyst. Using this method in warm conditions, the incorporation of 18 F gave yields of up to 92%. With automation for a 20 min reaction at 130 °C, a decrease in efficiency was observed but unlike the use of iodonium salt, the yield remained satisfying (40%, decay corrected yield). Using the latter method, with a view to preclinical evaluation, fully automated production was achieved in 75 min with a molar activity of 90 GBq/µmole on average.

### In vivo studies

The potential of the radiolabelled derivative of compound **16**, [^18^F]**16,** to image COX-2 by PET imaging was evaluated in a rodent model of neuroinflammation. This model consists of a unilateral intrastriatal injection of quinolinic acid to induce excitotoxicity mediated via NMDA receptor activation. This animal model is well known in the literature to produce an inflammatory reaction induced by COX-2 activation in lesioned cerebral parenchyma^23–25^.

**Figure 3.**
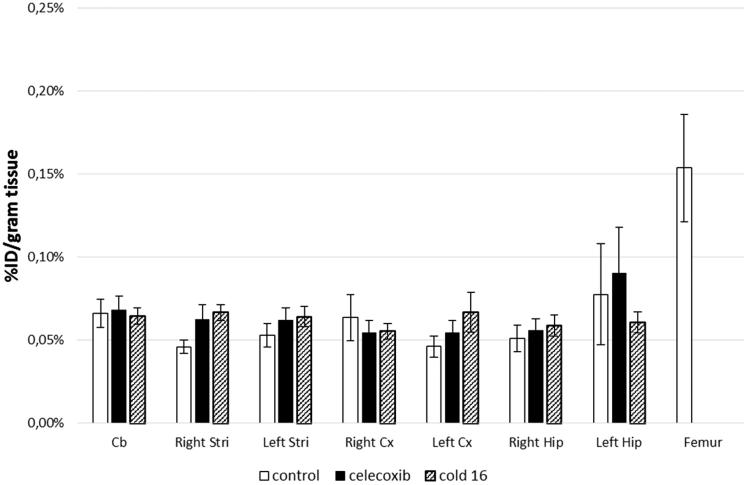
Cerebral biodistribution of **[**^18^**F]16**, 60 min after i.v. injection in rats, 7 days after focal intrastriatal injection of 150 nmol of quinolinic acid. In the “celecoxib” and “cold 16” groups, animals received an i.v. pre-injection (15 min) of the corresponding compound (3 mg/kg). Results are expressed as % injected dose/g tissue ± SEM. Cx: cortex; Stri: striatum; Hip: hippocampus, Cb: cerebellum.

The cerebral biodistribution of [^18^F]**16** in rats lesioned with QA was expressed as percent of injected dose per gram of tissue (%ID/g) and is shown in Figure 3. For the control group, the cerebral accumulation of [^18^F]**16** in non-activated brain regions one hour after i.v. injection was low (0.046 ± 0.004 to 0.078 ± 0.030%ID/g tissue). Furthermore, in activated brain areas, similar accumulations were found whatever the cerebral regions (cerebellum, striatum, cortex, hippocampus), with no statistically significant difference compared to the contralateral striatum and cortex, the brain areas that are the most affected by the neuroinflammatory process.

These preliminary results suggest a low passage of [^18^F]**16** through the blood-brain-barrier (BBB), even if the hypothesis of a fast cerebral kinetics of the tracer, characterised by a rapid peripheral release, could be investigated using *in vivo* micro-PET. Blocking *in vivo* experiments, consisting of a pretreatment with either celecoxib or cold **16** (3 mg/kg) 15 min before injection of the radiotracer, are also presented in Figure 3. In none of these experiments was a decrease in [^18^F]**16** accumulation observed. This confirmed that the low *in vivo* accumulation observed consisted of nonspecific binding. Finally, we observed a slight [^18^F]**16** bone uptake in the femur (0.154 ± 0.032%ID/g tissue), suggesting negligible defluorination *in vivo*.

The low brain uptake of [^18^F]**16** might be related to its rather low binding kinetic constant to the enzyme combined to a poor brain uptake. Recent attempts to develop an adequate COX-2 PET tracer faced the same unresolved issue at the pre-clinical evaluation step^26^^,^^27^. Therefore, whereas numerous COX-2 inhibitors have been proposed as therapeutic applications to modulate inflammation, there is still to date a lack of effective radiopharmaceutical drug to explore COX-2 by *in vivo* brain molecular imaging but other applications could be envisioned in cancer models^11^^,^^12^^,^^28^.

## Conclusion

The aim of the present study was to develop a series of novel derivatives exhibiting high affinity and selectivity towards the COX-2 enzyme. A straightforward synthesis was achieved, involving a bromination/arylation sequence under microwave irradiation in the indazole series, whereas in the azaindazole series direct C–H activation was achieved. The newly designed library was evaluated by an enzymatic cyclooxygenase inhibition assay *in vitro* to assess the inhibitory potencies of COX-2 and COX-1 peroxydases and SAR was discussed. The most promising compound **16** was then radiolabeled with fluorine 18 using a very interesting boronic ester displacement and the [^18^F]**16** evaluated as a tracer *in vivo* in a rat model of neuroinflammation. Although showing a submicromolar affinity for COX-2 and an excellent selectivity versus COX-1, cerebral biodistribution studies using [^18^F]**16** revealed a low brain penetrance combined with poor accumulation specificity associated with neuroinflammation. Because of its good *in vitro* characteristics; this radioligand deserves to be evaluated in peripheral inflammation and cancer animal models. In parallel, efforts are currently in progress regarding other original series to work around these limitations and improve the brain availability of new PET candidates. This work gives promising first results as a molecular scaffold able to inhibit COX-2 activity at the sub-micromolar level. However, more work is needed to explore this scaffold to increase the enzyme activity as well as to enhance its brain penetrance.

## Supplementary Material

Supporting_Information_J_Enz_Inhib_Med_Chem_Routier_revised_.docx
