# Supplementary material for: Design of selective COX-2 inhibitors in the (aza)indazole series. Chemistry, in vitro studies, radiochemistry and evaluations in rats of a [18F] PET tracer
Source: J Enzyme Inhib Med Chem. 2018 Oct 26;34(1):1–7. doi: 10.1080/14756366.2018.1501043 (PMC6211253; doi:10.1080/14756366.2018.1501043)
Supplement: Supporting_Information_J_Enz_Inhib_Med_Chem_Routier_revised_.docx [file IENZ_A_1501043_SM7987.docx]

**Supporting Information**

Design of selective COX-2 inhibitors in the (aza)indazole series. Chemistry, in vitro studies, radiochemistry and evaluations in rats of a [^18^F] PET tracer.

Jonathan Elie,^a,b^ Johnny Vercouillie,^b,c,d,e,g^ Nicolas Arlicot^,b,c,d,e,g,*^ Lucas Lemaire,^a^ Rudy Bidault,^b^ Sylvie Bodard,^b^ Christel Hosselet,^b,c^ Jean-Bernard Deloye,^c,f^ Sylvie Chalon, ^b^ Patrick Emond,^b^ Denis Guilloteau,^b,c,d,e^ Frédéric Buron,^a,*^ and Sylvain Routier^a,*^

^a^ ICOA, UMR CNRS 7311, University of Orleans, Orleans, France; ^b^ UMR Inserm U930, University of François Rabelais de Tours, Tours, France ; ^c^ CERRP, Centre d’Etude et de Recherche sur les Radiopharmaceutiques, Tours, France ; ^d^ CHRU de Tours, Tours, France ; ^e^ INSERM CIC 1415, University of François-Rabelais de Tours, Tours, France ; ^f^ Laboratoires Cyclopharma, Biopôle Clermont-Limagne, 63360 Saint-Beauzire, France ; ^g^ These authors contributed equally to the project.

The supporting information contains the materials, methods, and the ^1^H NMR and ^13^C NMR copy spectra.

# Materials and methods

## Chemistry-general

^1^H NMR and ^13^C NMR spectra were recorded on a Bruker DPX 250 MHz or 400 MHz instrument using CDCl_3_ or DMSO-*d_6_*. The chemical shifts are reported in parts per million (δ scale) and all coupling constant (*J*) values are in Hertz (Hz). The following abbreviations were used to explain the multiplicities: s (singlet), d (doublet), t (triplet), q (quartet), m (multiplet) and dd (doublet doublet). Melting points (m.p.) were determined using a Kofler hot-stage apparatus. IR absorption spectra were obtained on a Perkin Elmer PARAGON 1000 PC and values are reported in cm^-1^. HRMS were recorded on a Bruker maXis mass spectrometer by the "Federation de Recherche" ICOA/CBM (FR2708) platform. Monitoring of the reactions was performed using silica gel TLC plates (silica Merck 60 F254). Spots were visualized by UV light at 254 nm and 356 nm. Column chromatographies were performed using silica gel 60 (0.063-0.200 mm, Merck). Microwave irradiation was carried out in sealed 2-5 mL vessels placed in a Biotage Initiator system using a standard absorbance level (300 W maximum power). The temperatures were measured externally by an IR probe that determined the temperature on the surface of the vial and could be read directly from the instrument screen. The reaction time was measured once the reaction mixture had reached the stated temperature for temperature-controlled experiments. Pressure was measured by a non-invasive sensor integrated into the cavity.

## Synthesis

***General procedure A for imine synthesis.***

A microwave vial (2-5 mL) with a stir bar was charged with a mixture of 2-bromobenzaldehyde (0.200 g, 1.30 mmol, 1.0 eq.), dry THF (3 mL) and anhydrous MgSO_4_ (0.782 g, 6.50 mmol, 5.00 eq.). The mixture was stirred 2 min at room temperature and then the corresponding aniline (1.33 mmol, 1.01 eq.) was added. The vial was sealed and then placed in the microwave cavity. After the required reaction time of irradiation at 100 °C, the reaction mixture was cooled to room temperature and filtered. The filtrate was concentrated under vacuum to give **1-4, 28-31.**

***General procedure B for (aza)indazole formation.***

A microwave vial (2-5 mL) with a stir bar was charged with a mixture of the corresponding imine **1-3, 9, 28-31** (1.33 mmol), CuI (0.1 equiv), NaN_3_ (2.5 equiv) and dry DMSO (3 mL). The vial was sealed and then placed in the microwave cavity. After irradiation at 150 °C, the reaction mixture was cooled and then poured into cold water and stirred 5 min. The precipitate was filtered off, washed with cold water and then taken up with EtOAc. The filtrate was dried over MgSO_4_, concentrated and purified by flash chromatography to give the compounds **5,6, 32-35**.

***General procedure C for C-3 bromination.***

To a suspension of **5, 7**, **8**, **32, 34, 35** (1.0 equiv) in a mixture of AcOH/MeOH/DCM (2/1/0.1) was added Br_2_ (1.0 equiv) dropwise and then stirred 4 h at room temperature. The solvent was removed and then the crude product taken off with cold water, the solid was filtered off, washed successively with a saturated aqueous NaHCO_3_ solution, a saturated aqueous Na_2_SO_4_ solution, cold water and finally with Et_2_O. The solvent was removed under vacuum to give the pure compounds **11-13**, **36-38**.

***General procedure D for the C-3 Suzuki-Miyaura reaction.***

A microwave vial (2-5 mL) with a stir bar was charged with the bromo derivative **11-13** (1.00 equiv), the corresponding boronic acid (1.20 equiv), K_2_CO_3_ (3.0 equiv) and dioxane/water 9/1. The mixture was degassed 15 min and then Pd(PPh_3_)_4_ (0.10 equiv) was added. The vial was sealed and then placed in the microwave cavity. After 1 h of irradiation at 150 °C, the mixture was concentrated and purified by flash chromatography to give compounds **14-27**.

***1-(2-Bromophenyl)-N-(4-fluorophenyl)methanimine (1).***

Compound **1** was obtained following general procedure A using 4-fluoroaniline and 10 min of irradiation as a yellow solid in a 99% yield. Mp : 55-57 °C. IR (ATR diamond, cm^-1^) ν : 1616, 1498, 1267, 1230, 1215, 1187, 1147, 1023, 828, 776, 752, 717, 671. ^1^H NMR (250 MHz, CDCl_3_) δ : 7.04-7.15 (m, 2H, 2xH_Ar_), 7.17-7.32 (m, 2H, 2xH_Ar_), 7.32 (ddd, *J* = 7.3, 2.0 Hz, 1H, H_Ar_), 7.41 (dddd, *J* = 7.8, 7.3, 1.4, 0.7 Hz, 1H, H_Ar_), 7.59-7.67 (m, 1H, H_Ar_), 8.17-8.25 (m, 1H, H_Ar_), 8.84 (s, 1H, H_Imi_). ^13^C NMR (101 MHz, CDCl_3_) δ : 116.1 (d, *J* = 22.6 Hz, 2xCH_Ar_), 122.7 (d, *J* = 8.3 Hz, 2xCH_Ar_), 126.2 (C_q_), 127.90 (CH_Ar_), 129.1 (CH_Ar_) , 132.6 (CH_Ar_), 133.4 (CH_Ar_), 134.6 (C_q_), 147.8 (d, *J* = 3.0 Hz, C_q_), 159.1 (d, *J* = 1.8 Hz, CH_Imi_), 161.7 (d, *J* = 245.4 Hz, C_q_). ^19^F NMR (376 MHz, CDCl_3_) δ : -116.54 (CF). HRMS (EI-MS) : m/z calcd for C_13_H_9_BrFN : 277.9975 [M+H]^+^, found : 277.9971.

***1-(2-Bromophenyl)-N-(4-methylsulfanylphenyl)methanimine (2).***

Compound **2** was obtained following general procedure A using 4-(methylthio)aniline and 15 min of irradiation as a yellow solid in a 99% yield. Mp : 62-64 °C. IR (ATR diamond, cm^-1^) ν : 2911, 1613, 1489, 1438, 1192, 1903, 1025, 960, 810, 750. ^1^H NMR (400 MHz, CDCl_3_) δ : 2.52 (s, 3H, CH_3_), 7.21-7.25 (m, 2H, 2xH_Ar_), 7.29-7.34 (m, 3H, 3xH_Ar_), 7.38-7.43 (m, 1H, H_Ar_), 7.62 (dd, *J* = 7.9, 1.2 Hz, 1H, H_Ar_), 8.22 (dd, *J* = 7.9, 1.8 Hz, 1H, H_Ar_), 8.87 (s, 1H, H_Imi_). ^13^C NMR (101 MHz, CDCl_3_) δ : 16.4 (CH_3_), 121.9 (2xCH_Ar_), 126.1 (C_q_), 127.7 (2xCH_Ar_), 127.8 (CH_Ar_), 129.1 (CH_Ar_), 132.4 (CH_Ar_), 133.3 (CH_Ar_), 134.7 (C_q_), 136.7 (C_q_), 148.9 (C_q_), 158.6 (CH_Imi_). HRMS (EI-MS) : m/z calcd for C_14_H_12_BrNS : 305.9946 [M+H]^+^, found : 305.9949.

***1-(2-Bromophenyl)-N-(4-methylsulfonylphenyl)methanimine (3).***

Compound **3** was obtained following general procedure A using 4-methylsulfonylaniline and 1.5 h of irradiation as a yellow solid in a 95% yield. Mp : 119-121 °C. IR (ATR diamond, cm^-1^) ν : 1581, 1290, 1188, 1136, 1083, 1023, 960, 836, 758, 722. ^1^H NMR (400 MHz, CDCl_3_) δ : 3.08 (s, 3H, CH_3_), 7.32 (dd, *J* = 8.3, 1.5 Hz, 2H, 2xH_Ar_), 7.35-7.40 (m, 1H, H_Ar_), 7.44 (t, *J* = 7.5 Hz, 1H, H_Ar_), 7.65 (d, *J* = 7.9 Hz, 1H, H_Ar_), 7.98 (dd, *J* = 8.3, 1.5 Hz, 2H, 2xH_Ar_), 8.22 (dt, *J* = 7.9, 1.5 Hz, 1H, H_Ar_), 8.82 (s, 1H, H_Imi_). ^13^C NMR (101 MHz, CDCl_3_) δ : 44.8 (CH_3_), 121.7 (2xCH_Ar_), 126.6 (C_q_), 128.0 (CH_Imi_), 128.9 (2xCH_Ar_), 129.4 (CH_Ar_), 133.4 (CH_Ar_), 133.5 (CH_Ar_), 133.9 (C_q_), 137.7 (C_q_), 156.7 (C_q_), 161.8 (C_q_). HRMS (EI-MS) : m/z calcd for C_14_H_12_BrNO_2_S : 337.9845 [M+H]^+^, found : 337.9840.

***4-[(2-Bromophenyl)methyleneamino]benzenesulfonamide (4).***

Compound **4** was obtained following general procedure A using 4-aminobenzenesulfonamide and 1.5 h of irradiation as a white solid in a 96% yield. Mp : 174-176 °C. IR (ATR diamond, cm^-1^) ν : 3293, 3054, 1613, 1580, 1561, 1338, 1155, 1092, 842, 827, 750, 718. ^1^H NMR (250 MHz, DMSO-*d*_6_) δ : 7.38 (s, 2H, NH_2_), 7.39-7.45 (m, 2H, 2xH_Ar_), 7.48-7.58 (m, 2H, 2xH_Ar_), 7.76-7.83 (m, 1H, H_Ar_), 7.85-7.92 (m, 2H, 2xH_Ar_), 8.13-8.20 (m, 1H, H_Ar_), 8.79 (s, 1H, H_Imi_). ^13^C NMR (63 MHz, DMSO-*d*_6_) δ : 121.2 (2xCH_Ar_), 125.5 (CH_Ar_), 127.0 (2xCH_Ar_), 128.2 (CH_Ar_), 129.0 (CH_Ar_), 133.4 (CH_Ar_), 133.5 (C_q_), 133.6 (CH_Ar_), 141.6 (C_q_), 153.9 (C_q_), 160.7 (CH_Imi_). HRMS (EI-MS) : m/z calcd for C_13_H_11_BrN_2_O_2_S : 338.9797 [M+H]^+^, found : 338.9797.

***N'-[4-[(2-Bromophenyl)methyleneamino]phenyl]sulfonyl-N,N-dimethyl-formamidine (9).***

To a solution of **4** (13.8 g, 40.7 mmol, 1.0 eq.) in dry THF (200 mL) was added DME.DMA (11.23 mL, 81.4 mmol, 2.0 eq.). The mixture was stirred 1 h at room temperature. Then the solvent was removed and the residue was triturated in Et_2_O (30 mL), filtered off and dried under vacuum to give the pure title compound **13** (15.76 g, 98 %) as a pale yellow solid. Mp : 145-147 °C. IR (ATR diamond, cm^-1^) ν : 1616, 1430, 1345, 1287, 1140, 1081, 898, 847, 837, 747, 719, 663, 621. ^1^H NMR (250 MHz, CDCl_3_) δ : 3.04 (s, 3H, CH_3_), 3.14 (s, 3H, CH_3_), 7.18-7.31 (m, 2H, 2xH_Ar_), 7.31-7.38 (m, 1H, H_Ar_), 7.42 (tdd, *J* = 7.8, 1.6, 0.7 Hz, 1H, H_Ar_), 7.60-7.65 (m, 1H, H_Ar_), 7.88-7.96 (m, 2H, 2xH_Ar_), 8.16 (d, *J* = 0.7 Hz, 1H, H_Ar_), 8.17-8.24 (m, 1H, H_Imi_), 8.80 (s, 1H, H_Imi_). ^13^C NMR (101 MHz, CDCl_3_) δ : 35.6 (CH_3_), 41.6 (CH_3_), 121.2 (2xCH_Ar_), 126.4 (C_q_), 127.9 (2xCH_Ar_), 127.9 (CH_Ar_), 129.3 (CH_Ar_), 133.1 (CH_Ar_), 133.4 (CH_Ar_), 134.1 (C_q_), 139.9 (C_q_), 155.0 (C_q_), 159.2 (CH_Imi_), 161.1 (CH_Imi_). HRMS (EI-MS) : m/z calcd for C_16_H_16_BrN_3_O_2_S : 394.0219 [M+H]^+^, found : 394.0217.

***2-(4-Fluorophenyl)indazole (5).***

Compound **5** was obtained following general procedure B using derivative **1** as starting material. The irradiation time was 5 min and purification was performed using Petroleum Ether/EtOAc (7/3) as eluent to furnish **5** as a yellow solid in a 65% yield. **Rf** (Petroleum Ether/EtOAc, 5/5) : 0.82. Mp : 105-107 °C. IR (ATR diamond, cm^-1^) ν : 1631, 1517, 1506, 1383, 1292, 1226, 1202, 1153, 1097, 1036, 951, 838, 816, 779, 750, 728. ^1^H NMR (250 MHz, CDCl_3_) δ : 7.12 (ddd, *J* = 8.5, 6.6, 1.0 Hz, 1H, H_Ar_), 7.17-7.27 (m, 2H, 2xH_Ar_), 7.33 (ddd, *J* = 8.8, 6.6, 1.0 Hz, 1H, H_Ar_), 7.71 (dt, *J* = 8.5, 1.0 Hz, 1H, H_Ar_), 7.78 (d_q_, *J* = 8.8, 1.0 Hz, 1H, H_Ar_), 7.83-7.92 (m, 2H, 2xH_Ar_), 8.35 (d, *J* = 1.0 Hz, 1H, H_Ar_). ^13^C NMR (63 MHz, CDCl_3_) δ : 116.6 (d, *J* = 23.1 Hz, 2xCH_Ar_), 118.0 (CH_Ar_), 120.1 (C_q_), 120.4 (CH_Ar_), 120.6 (CH_Ar_), 122.8 (2xd, *J* = 7.5 Hz, CH_Ar_), 122.9 (CH_Ar_), 127.0 (CH_Ar_), 137.0 (d, *J* = 2.1 Hz, C_q_), 149.9 (C_q_), 162.1 (d, *J* = 248.0 Hz, C_q_). ^19^F NMR (235 MHz, CDCl_3_) δ : -113.82 (CF). HRMS (EI-MS) : m/z calcd for C_13_H_9_FN_2_: 213.0822 [M+H]^+^, found : 213.0820.

***2-(4-Methylsulfanylphenyl)indazole (6).***

Compound **6** was obtained following general procedure B using derivative **2** as starting material. The irradiation time was 5 min and the purification was performed using Petroleum Ether/EtOAc (9/1 then 7/3) as eluent to furnish **6** as a yellow solid in a 56% yield. Rf (Petroleum Ether/EtOAc, 7/3) : 0.57. Mp : 139-141 °C. IR (ATR diamond, cm^-1^) ν : 1628, 1516, 1497, 1417, 1380, 1343, 1299, 1207, 1147, 1125, 1096, 1044, 950, 836, 807, 777, 750. ^1^H NMR (400 MHz, CDCl_3_) δ : 2.54 (s, 3H, CH_3_), 7.08-7.15 (m, 1H, H_Ar_), 7.29-7.35 (m, 1H, H_Ar_), 7.36-7.42 (m, 2H, 2xH_Ar_), 7.70 (dt, *J* = 8.7, 1.1 Hz, 1H, H_Ar_), 7.78 (d, *J* = 8.7 Hz, 1H, H_Ar_), 7.81-7.86 (m, 2H, 2xH_Ar_), 8.37 (s, 1H, H_Ar_). ^13^C NMR (101 MHz, CDCl_3_) δ : 16.0 (CH_3_), 118.0 (CH_Ar_), 120.2 (CH_Ar_), 120.4 (CH_Ar_), 121.4 (2xCH_Ar_), 122.6 (CH_Ar_), 122.9 (C_q_), 126.9 (CH_Ar_), 127.4 (2xCH_Ar_), 137.9 (C_q_), 138.7 (C_q_), 149.8 (C_q_). HRMS (EI-MS) : m/z calcd for C_14_H_12_N_2_S : 241.0794 [M+H]^+^, found : 241.0795.

***2-(4-Methylsulfonylphenyl)indazole (7).***

Compound **7** was obtained following general procedure B using derivative **3** as starting material. The irradiation time was 5 min and the purification was performed using Petroleum Ether/EtOAc (5/5) as eluent to furnish **7** as a white solid in a 17% yield. Alternatively, the same derivative was obtained as follows. To a solution of **6** (600 mg, 2.50 mmol) in MeOH/H_2_O (1/1, 140 mL) was added Oxone (1.614 g, 5.25 mmol, 2.1 equiv). The mixture was stirred 20 h at room temperature. Volatiles were partially removed under vacuum and then taken off in CH_2_Cl_2_ (50 mL) and water (75 mL). The aqueous layer was extracted twice with CH_2_Cl_2_ (50 mL). Organic layers were combined, dried over MgSO_4_, filtered, concentrated under vacuum and the crude product was purified by flash chromatography with Petroleum Ether/EtOAc (6/4 then 5/5) to give **6** (0.568 g) in a 83% yield. **Rf** (Petroleum Ether/EtOAc, 5/5) : 0.51. Mp : 199-201 °C. IR (ATR diamond, cm^-1^) ν : 1592, 1515, 1422, 1379, 1295, 1204, 1144, 1092, 1039, 967, 948, 850, 820, 806, 777, 749, 723. ^1^H NMR (400 MHz, DMSO-*d*_6_) δ : 3.30 (s, 3H, CH_3_), 7.14 (dd, *J* = 8.6, 6.5 Hz, 1H, H_Ar_), 7.36 (dd, *J* = 8.8, 6.5 Hz, 1H, H_Ar_), 7.73 (d, *J* = 8.7 Hz, 1H, H_Ar_), 7.79 (d, *J* = 8.7 Hz, 1H, H_Ar_), 8.11-8.17 (m, 2H, 2xH_Ar_), 8.37-8.42 (m, 2H, 2xH_Ar_), 9.27 (s, 1H, H_Ar_). ^13^C NMR (101 MHz, DMSO-*d*_6_) δ : 43.5 (CH_3_), 117.6 (CH_Ar_), 120.5 (2xCH_Ar_), 121.1 (CH_Ar_), 122.5 (CH_Ar_), 122.6 (C_q_), 122.7 (CH_Ar_), 127.6 (CH_Ar_), 128.8 (2xCH_Ar_), 139.4 (C_q_), 143.2 (C_q_), 149.46 (C_q_).HRMS (EI-MS) : m/z calcd for C_14_H_12_N_2_O_2_S : 273.0692 [M+H]^+^, found : 273.0695.

***4-Indazol-2-ylbenzenesulfonamide (8).***

Compound **8** was obtained following general procedure B using derivative **9** as starting material. The irradiation time was 5 min. After completion of the reaction, NaOH (5.496 g, 137.40 mmol, 3.0 equiv) was added and stirred 5 min at 150 °C. After cooling, the mixture was poured into cold water (240 mL) and the aqueous mixture was acidified with HCl 12N (pH ≤ 3). The resulting precipitate was filtered, washed with cold water and then taken off with a mixture of EtOAc/MeOH (8/2). The organic layer was dried over MgSO_4_, filtered, concentrated and precipitated in CH_2_Cl_2_. The solid was filtered off, washed with CH_2_Cl_2_ and dried under vacuum to give **8** (10.600 g, 85 % from **9**) as a brown solid. Mp : 251-253 °C. IR (ATR diamond, cm^-1^) ν : 3324, 2495, 1595, 1519, 1384, 1309, 1153, 1096, 899, 833, 817, 752. ^1^H NMR (400 MHz, DMSO-*d*_6_) δ : 7.10-7.17 (m, 1H, H_Ar_), 7.35 (ddd, *J* = 8.9, 6.6, 1.1 Hz, 1H, H_Ar_), 7.49 (s, 2H, NH_2_), 7.69-7.77 (m, 1H, H_Ar_), 7.79 (dt, *J* = 8.3, 1.1 Hz, 1H, H_Ar_), 7.96-8.06 (m, 2H, 2xH_Ar_), 8.27-8.35 (m, 2H, 2xH_Ar_), 9.21 (s, 1H, H_Ar_). ^13^C NMR (101 MHz, DMSO-*d*_6_) δ : 117.5 (CH_Ar_), 120.3 (2xCH_Ar_), 121.0 (CH_Ar_), 122.2 (CH_Ar_), 122.5 (CH_Ar_), 122.6 (C_q_), 127.3 (2xCH_Ar_), 127.4 (CH_Ar_), 141.9 (C_q_), 142.9 (C_q_), 149.3 (C_q_). HRMS (EI-MS) : m/z calcd for C_13_H_11_N_3_O_2_S: 274.0645 [M+H]^+^, found : 274.0647.

***3-Bromo-2-(4-fluorophenyl)indazole (11).***

Following general procedure C using **5** as starting material, compound **11** was obtained as a pale yellow solid in a 95% yield. Rf (Petroleum Ether/EtOAc, 95/5) : 0.28. Mp : 104-106 °C. IR (ATR diamond, cm^-1^) ν : 1603, 1505, 1359, 1324, 1226, 1212, 1151, 1092, 1006, 916, 836, 794, 754, 730. ^1^H NMR (250 MHz, CDCl_3_) δ : 7.14-7.30 (m, 3H, 3xH_Ar_), 7.37 (ddd, *J* = 8.8, 6.6, 1.2 Hz, 1H, H_Ar_), 7.58 (dt, *J* = 8.5, 1.0 Hz, 1H, H_Ar_), 7.61-7.70 (m, 2H, 2xH_Ar_), 7.73 (dt, *J* = 8.5, 1.0 Hz, 1H, H_Ar_). ^13^C NMR (63 MHz, CDCl_3_) δ : 106.4 (C_q_), 116.2 (d, *J* = 23.2 Hz, 2xCH_Ar_), 118.2 (CH_Ar_), 119.7 (CH_Ar_), 122.9 (C_q_), 123.2 (CH_Ar_), 127.8 (CH_Ar_), 128.2 (d, *J* = 8.9 Hz, 2xCH_Ar_), 135.4 (d, *J* = 3.3 Hz, C_q_), 149.3 (C_q_), 162.8 (d, *J* = 249.8 Hz, C_q_). ^19^F NMR (235 MHz, CDCl_3_) δ : -111.26 (CF). HRMS (EI-MS) : m/z calcd for C_13_H_8_BrFN_2_ : 290.9928 [M+H]^+^, found : 290.9926.

***3-Bromo-2-(4-methylsulfonylphenyl)indazole (12).***

Following general procedure C using **7** as starting material, compound **12** was obtained as a yellow solid in a 99% yield. Rf (Petroleum Ether/EtOAc, 5/5) : 0.38. Mp : 154-156 °C. IR (ATR diamond, cm^-1^) ν : 1594, 1505, 1367, 1292, 1141, 1096, 1086, 1002, 965, 839, 774, 756, 739. ^1^H NMR (400 MHz, CDCl_3_) δ : 3.13 (s, 3H, CH_3_), 7.19 (ddd, *J* = 8.5, 6.6, 0.9 Hz, 1H, H_Ar_), 7.38 (ddd, *J* = 8.9, 6.6, 1.1 Hz, 1H, H_Ar_), 7.57 (dt, *J* = 8.5, 1.0 Hz, 1H, H_Ar_), 7.72 (dt, *J* = 8.9, 0.9 Hz, 1H, H_Ar_), 7.95-8.01 (m, 2H, 2xH_Ar_), 8.11-8.16 (m, 2H, 2xH_Ar_). ^13^C NMR (101 MHz, CDCl_3_) δ : 44.6 (CH_3_), 106.1 (C_q_), 118.3 (CH_Ar_), 119.8 (CH_Ar_), 123.5 (C_q_), 123.8 (CH_Ar_), 126.9 (2xCH_Ar_), 128.4 (CH_Ar_), 128.6 (2xCH_Ar_), 140.9 (C_q_), 143.5 (C_q_), 149.8 (C_q_). HRMS (EI-MS) : m/z calcd for C_14_H_11_BrN_2_O_2_S : 350.9797 [M+H]^+^, found : 350.9797.

***4-(3-Bromoindazol-2-yl)benzenesulfonamide (13).***

Following general procedure C using **8** as starting material, compound **13** was obtained as a brown solid in a 99% yield. Mp : 237-239 °C. IR (ATR diamond, cm^-1^) ν : 3293, 1595, 1509, 1338, 1153, 1099, 1010, 916, 833, 746, 625. ^1^H NMR (400 MHz, DMSO-*d*_6_) δ : 7.24 (dd, *J* = 8.5, 6.5 Hz, 1H, H_Ar_), 7.43 (dd, *J* = 8.5, 6.5 Hz, 1H, H_Ar_), 7.60 (s, 2H, NH_2_), 7.63 (d, *J* = 8.8 Hz, 1H, H_Ar_), 7.75 (d, *J* = 8.8 Hz, 1H, H_Ar_), 7.94-8.11 (m, 4H, 4xH_Ar_). ^13^C NMR (101 MHz, DMSO-*d*_6_) δ : 106.9 (C_q_), 117.9 (CH_Ar_), 119.6 (CH_Ar_), 122.5 (C_q_), 123.3 (CH_Ar_), 126.7 (2xCH_Ar_), 126.7 (2xCH_Ar_), 128.0 (CH_Ar_), 141.0 (C_q_), 144.6 (C_q_), 148.7 (C_q_). HRMS (EI-MS) : m/z calcd for C_13_H_10_BrN_3_O_2_S: 351.9750 [M+H]^+^, found : 351.9748.

***3-(4-Fluorophenyl)-2-(4-methylsulfonylphenyl)indazole (14).***

Compound **14** was obtained following general procedure D using derivative **12** as starting material. Purification was performed using Petroleum Ether/EtOAc (5/5) as eluent to furnish **14** as a pale yellow solid in a 95% yield. Rf (Petroleum Ether/EtOAc, 5/5) : 0.31. Mp : 173-175 °C. IR (ATR diamond, cm^-1^) ν : 1590, 1506, 1403, 1356, 1315, 1296, 1219, 1149, 1090, 952, 850, 841, 772, 752, 722. ^1^H NMR (400 MHz, CDCl_3_) δ : 3.11 (s, 3H, CH_3_), 7.16-7.22 (m, 3H, 3xH_Ar_), 7.34-7.40 (m, 2H, 2xH_Ar_), 7.42 (ddt, *J* = 9.3, 6.7, 1.3 Hz, 1H, H_Ar_), 7.64-7.71 (m, 3H, 3xH_Ar_), 7.81 (dt, *J* = 8.8, 1.1 Hz, 1H, H_Ar_), 7.98-8.03 (m, 2H, 2xH_Ar_). ^13^C NMR (101 MHz, CDCl_3_) δ : 44.6 (CH_3_), 116.7 (d, *J* = 21.9 Hz, 2xCH_Ar_), 118.0 (CH_Ar_), 120.4 (CH_Ar_), 122.4 (C_q_), 123.5 (CH_Ar_), 125.5 (d, *J* = 3.7 Hz, C_q_), 126.5 (2xCH_Ar_), 128.0 (CH_Ar_), 128.6 (2xCH_Ar_), 131.6 (d, *J* = 8.3 Hz, 2xCH_Ar_), 134.9 (C_q_), 140.0 (C_q_), 144.4 (C_q_), 149.6 (C_q_), 163.0 (d, *J* = 250.6 Hz, C_q_). ^19^F NMR (376 MHz, CDCl_3_) δ : -111.01 (CF). HRMS (EI-MS) : m/z calcd for C_20_H_15_FN_2_O_2_S : 367.0911 [M+H]^+^, found : 367.0911.

***2-(4-Fluorophenyl)-3-(4-methylsulfonylphenyl)indazole (15).***

Compound **15** was obtained following general procedure D using derivative **11** as starting material. Purification was performed using Petroleum Ether/EtOAc (7/3 then 5/5) as eluent to furnish **15** as a white solid in a 47% yield. Rf (Petroleum Ether/EtOAc, 5/5) : 0.54. Mp : 198-200 °C. IR (ATR diamond, cm^-1^) ν : 1593, 1510, 1299, 1222, 1183, 1146, 1085, 956, 845, 800, 773, 749, 719, 675. ^1^H NMR (400 MHz, CDCl_3_) δ : 3.12 (s, 3H, CH_3_), 7.10-7.17 (m, 2H, 2xH_Ar_), 7.22 (ddd, *J* = 8.6, 6.6, 0.9 Hz, 1H, H_Ar_), 7.41 (ddd, *J* = 6.9, 5.6, 2.5 Hz, 3H, 3xH_Ar_), 7.53-7.58 (m, 2H, 2xH_Ar_), 7.70 (dt, *J* = 8.6, 1.0 Hz, 1H, H_Ar_), 7.83 (dt, *J* = 8.7, 0.9 Hz, 1H, H_Ar_), 7.96-8.02 (m, 2H, 2xH_Ar_). ^13^C NMR (101 MHz, CDCl_3_) δ : 44.5 (CH_3_), 116.6 (d, *J* = 23.1 Hz, 2xCH_Ar_), 118.3 (CH_Ar_), 119.7 (CH_Ar_), 122.1 (C_q_), 124.0 (CH_Ar_), 127.6 (CH_Ar_), 127.9 (d, *J* = 8.7 Hz, 2xCH_Ar_), 128.1 (2xCH_Ar_), 130.3 (2xCH_Ar_), 133.2 (C_q_), 135.3 (C_q_), 136.0 (d, *J* = 3.4 Hz, C_q_), 140.2 (C_q_), 149.2 (C_q_), 162.5 (d, *J* = 250.1 Hz, C_q_). ^19^F NMR (376 MHz, CDCl_3_) δ : -111.44 (CF). HRMS (EI-MS) : m/z calcd for C_20_H_15_FN_2_O_2_S : 367.0911 [M+H]^+^, found : 367.0910.

***4-[3-(4-Fluorophenyl)indazol-2-yl]benzenesulfonamide (16).***

Compound **16** was obtained following general procedure D using derivative **11** as starting material. Purification was performed using Petroleum Ether/EtOAc (5/5) as eluent to furnish **16** as a white solid in a 73% yield. Rf (Petroleum Ether/EtOAc, 5/5) : 0.40. Mp : 229-231 °C. IR (ATR diamond, cm^-1^) ν :: 3272, 3157, 1593, 1507, 1363, 1338, 1237, 1161, 1093, 838, 755, 738, 627. ^1^H NMR (400 MHz, DMSO-*d*_6_) δ : 7.18 (ddd, *J* = 8.6, 6.6, 0.9 Hz, 1H, H_Ar_), 7.32-7.38 (m, 2H, 2xH_Ar_), 7.41 (ddd, *J* = 8.8, 6.5, 1.1 Hz, 1H, H_Ar_), 7.44-7.50 (m, 2H, 2xH_Ar_), 7.52 (s, 2H, NH_2_), 7.63-7.68 (m, 3H, 3xH_Ar_), 7.76 (dt, *J* = 8.8, 1.0 Hz, 1H, H_Ar_), 7.87-7.94 (m, 2H, 2xH_Ar_). ^13^C NMR (101 MHz, DMSO-*d*_6_) δ : 116.2 (d, *J* = 21.8 Hz, 2xCH_Ar_), 117.4 (CH_Ar_), 120.3 (CH_Ar_), 121.5 (C_q_), 122.9 (CH_Ar_), 125.3 (d, *J* = 3.3 Hz, C_q_), 126.4 (2xCH_Ar_), 126.6 (2xCH_Ar_), 127.3 (CH_Ar_), 131.9 (d, *J* = 8.5 Hz, 2xCH_Ar_), 134.4 (C_q_), 141.9 (C_q_), 143.7 (C_q_), 148.4 (C_q_), 162.1 (d, *J* = 247.0 Hz, C_q_). ^19^F NMR (376 MHz, DMSO-*d*_6_) δ : -112.18 (CF). HRMS (EI-MS) : m/z calcd for C_19_H_14_FN_3_O_2_S : 368.0863 [M+H]^+^, found : 368.0863.

***4-[3-(3-Fluorophenyl)indazol-2-yl]benzenesulfonamide (17).***

Compound **17** was obtained following general procedure D using derivative **13** as starting material. Purification was performed using Petroleum Ether/EtOAc (5/5) as eluent to furnish **17** as a white solid in a 59% yield. Rf (Petroleum Ether/EtOAc, 5/5) : 0.37. Mp : > 250 °C. IR (ATR diamond, cm^-1^) ν : 3300, 3132, 1593, 1505, 1362, 1338, 1326, 1227, 1162, 1101, 1011, 912, 879, 863, 833, 790, 758, 739, 690. ^1^H NMR (250 MHz, DMSO-*d*_6_) δ : 7.16-7.25 (m, 2H, 2xH_Ar_), 7.26-7.36 (m, 2H, 2xH_Ar_), 7.42 (ddd, *J* = 8.8, 6.6, 1.1 Hz, 1H, H_Ar_), 7.48-7.59 (m, 3H, NH_2_ & H_Ar_), 7.63-7.73 (m, 3H, 3xH_Ar_), 7.78 (dt, *J* = 8.8, 1.0 Hz, 1H, H_Ar_), 7.88-7.95 (m, 2H, 2xH_Ar_). ^13^C NMR (63 MHz, DMSO-*d*_6_) δ : 116.0 (d, *J* = 21.9 Hz, CH_Ar_), 115.8 (C_q_), 116.1 (d, *J* = 64.9 Hz, CH_Ar_), 117.5 (CH_Ar_), 120.2 (CH_Ar_), 121.5 (C_q_), 123.1 (CH_Ar_), 125.9 (d, *J* = 2.5 Hz, CH_Ar_), 126.4 (2xCH_Ar_), 126.6 (2xCH_Ar_), 127.4 (CH_Ar_), 131.0 (d, *J* = 8.4 Hz, C_q_), 131.1 (d, *J* = 8.1 Hz, CH_Ar_), 133.9 (d, *J* = 2.4 Hz, C_q_), 141.8 (C_q_), 143.8 (C_q_), 148.4 (C_q_). ^19^F NMR (235 MHz, DMSO-*d*_6_) δ : -111.76 (CF). HRMS (EI-MS) : m/z calcd for C_19_H_14_FN_3_O_2_S : 368.0863 [M+H]^+^, found : 368.0863.

***3-(3-Fluoro-4-methyl-phenyl)-2-(4-methylsulfonylphenyl)indazole (18).***

Compound **18** was obtained following general procedure D using derivative **12** as starting material. Purification was performed using Petroleum Ether/EtOAc (5/5) as eluent to furnish **18** as a yellow solid in a 95% yield. Rf (Petroleum Ether/EtOAc, 5/5) : 0.46. Mp : 149-151 °C. IR (ATR diamond, cm^-1^) ν : 1592, 1505, 1361, 1361, 1294, 1216, 1147, 1132, 1090, 954, 877, 842, 774, 745, 725. ^1^H NMR (400 MHz, CDCl_3_) δ : 2.35 (s, 3H, CH_3_), 3.09 (s, 3H, CH_3_), 6.98 (d, *J* = 7.8 Hz, 1H, H_Ar_), 7.06 (d, *J* = 10.0 Hz, 1H, H_Ar_), 7.17 (t, *J* = 7.6 Hz, 1H, H_Ar_), 7.25 (t, *J* = 7.9 Hz, 1H, H_Ar_), 7.39 (t, *J* = 7.7 Hz, 1H, H_Ar_), 7.62-7.72 (m, 3H, 3xH_Ar_), 7.77 (d, *J* = 8.8 Hz, 1H, H_Ar_), 7.98 (d, *J* = 8.3 Hz, 2H, 2xH_Ar_). ^13^C NMR (101 MHz, CDCl_3_) δ : 14.6 (d, *J* = 3.4 Hz, CH_3_), 44.7 (CH_3_), 116.2 (d, *J* = 23.7 Hz, CH_Ar_), 118.0 (CH_Ar_), 120.4 (CH_Ar_), 122.4 (C_q_), 123.5 (CH_Ar_), 125.3 (d, *J* = 3.5 Hz, CH_Ar_), 126.2 (d, *J* = 17.1 Hz, C_q_), 126.5 (2xCH_Ar_), 128.0 (CH_Ar_), 128.5 (d, *J* = 8.4 Hz, C_q_), 128.6 (2xCH_Ar_), 132.5 (d, *J* = 5.6 Hz, CH_Ar_), 134.8 (d, *J* = 2.2 Hz, C_q_), 140.0 (C_q_), 144.5 (C_q_), 149.6 (C_q_), 161.5 (d, *J* = 247.1 Hz, C_q_). ^19^F NMR (376 MHz, CDCl_3_) δ : -115.19 (CF). HRMS (EI-MS) : m/z calcd for C_21_H_17_FN_2_O_2_S : 381.1067 [M+H]^+^, found : 381.1068.

***3-(4-Ethoxy-3-fluoro-phenyl)-2-(4-methylsulfonylphenyl)indazole (19).***

Compound **19** was obtained following general procedure D using derivative **12** as starting material. Purification was performed using Petroleum Ether/EtOAc (5/5) as eluent to furnish **19** as a white solid in a 98% yield. Rf (Petroleum Ether/EtOAc, 5/5) : 0.37. Mp : 138-140 °C. IR (ATR diamond, cm^-1^) ν : 2977, 1600, 1504, 1469, 1394, 1364, 1313, 1297, 1272, 1219, 1148, 1133, 1092, 1038, 954, 871, 842, 776, 757, 720. ^1^H NMR (400 MHz, CDCl_3_) δ : 1.50 (t, *J* = 7.0 Hz, 3H, CH_3_), 3.09 (s, 3H, CH_3_), 4.17 (q, *J* = 6.9 Hz, 2H, CH_2_), 6.97-7.04 (m, 2H, 2xH_Ar_), 7.10-7.20 (m, 2H, 2xH_Ar_), 7.36-7.42 (m, 1H, H_Ar_), 7.67 (dd, *J* = 11.6, 8.3 Hz, 3H, 3xH_Ar_), 7.77 (d, *J* = 8.8 Hz, 1H, H_Ar_), 7.95-8.02 (m, 2H, 2xH_Ar_). ^13^C NMR (101 MHz, CDCl_3_) δ : 14.7 (CH_3_), 44.5 (CH_3_), 64.9 (CH_2_), 114.8 (d, *J* = 2.6 Hz, CH_Ar_), 117.3 (d, *J* = 19.7 Hz, CH_Ar_), 117.8 (CH_Ar_), 120.3 (CH_Ar_), 121.6 (d, *J* = 7.1 Hz, C_q_), 122.2 (C_q_), 123.3 (CH_Ar_), 125.9 (d, *J* = 3.5 Hz, CH_Ar_), 126.4 (2xCH_Ar_), 127.8 (CH_Ar_), 128.5 (2xCH_Ar_), 134.6 (d, *J* = 2.0 Hz, C_q_), 139.8 (C_q_), 144.4 (C_q_), 147.6 (C_q_), 149.5 (d, *J* = 348.3 Hz, C_q_), 149.5 (C_q_). ^19^F NMR (376 MHz, CDCl_3_) δ : -132.51 (CF). HRMS (EI-MS) : m/z calcd for C_22_H_19_FN_2_O_3_S : 411.1173 [M+H]^+^, found : 411.1177.

***3-[4-Ethoxy-3-(trifluoromethyl)phenyl]-2-(4-methylsulfonylphenyl)indazole (20).***

Compound **20** was obtained following general procedure D using derivative **12** as starting material. Purification was performed using Petroleum Ether/EtOAc (5/5) as eluent to furnish **20** as a white solid in a 72% yield. Rf (Petroleum Ether/EtOAc, 5/5) : 0.37. Mp : 196-198 °C. IR (ATR diamond, cm^-1^) ν : 3012, 2929, 1622, 1590, 1502, 1397, 1359, 1313, 1285, 1257, 1182, 1145, 1130, 1086, 1054, 952, 908, 853, 778, 762, 687. ^1^H NMR (400 MHz, CDCl_3_) δ : 1.49 (t, *J* = 7.0 Hz, 3H, CH_3_), 3.08 (d, *J* = 0.9 Hz, 3H, CH_3_), 4.18 (q, *J* = 7.0 Hz, 2H, CH_2_), 7.03 (d, *J* = 8.6 Hz, 1H, H_Ar_), 7.18 (ddt, *J* = 8.5, 6.6, 1.0 Hz, 1H, H_Ar_), 7.34 (dd, *J* = 8.7, 2.2 Hz, 1H, H_Ar_), 7.40 (ddt, *J* = 8.6, 6.6, 1.0 Hz, 1H, H_Ar_), 7.61-7.72 (m, 4H, 4xH_Ar_), 7.78 (dt, *J* = 8.8, 1.0 Hz, 1H, H_Ar_), 7.96-8.05 (m, 2H, 2xH_Ar_). ^13^C NMR (101 MHz, CDCl_3_) δ : 14.6 (CH_3_), 44.7 (CH_3_), 64.9 (CH_2_), 113.7 (CH_Ar_), 118.1 (CH_Ar_), 119.9 (d, *J* = 31.5 Hz, C_q_), 120.2 (CH_Ar_), 121.0 (C_q_), 122.3 (C_q_), 123.6 (CH_Ar_), 124.5 (d, *J* = 8.0 Hz, C_q_), 126.7 (2xCH_Ar_), 128.0 (CH_Ar_), 128.4 (q, *J* = 5.2 Hz, CH_Ar_), 128.7 (2xCH_Ar_), 134.5 (C_q_), 134.6 (C_q_), 140.1 (C_q_), 144.4 (C_q_), 149.7 (C_q_), 157.4 (C_q_). ^19^F NMR (376 MHz, CDCl_3_) δ : -62.74 (CF_3_). HRMS (EI-MS) : m/z calcd for C_23_H_19_F_3_N_2_O_3_S : 461.1141 [M+H]^+^, found : 461.1141.

***3-[4-Chloro-3-(trifluoromethyl)phenyl]-2-(4-methylsulfonylphenyl)indazole (21).***

Compound **21** was obtained following general procedure D using derivative **12** as starting material. Purification was performed using Petroleum Ether/EtOAc (5/5) as eluent to furnish **21** as a white solid in a 91% yield. Rf (Petroleum Ether/EtOAc, 5/5) : 0.46. Mp : 197-199 °C. IR (ATR diamond, cm^-1^) ν : 1587, 1498, 1359, 1310, 1298, 1175, 1144, 1109, 1035, 947, 845, 772, 748, 729, 670. ^1^H NMR (400 MHz, CDCl_3_) δ : 3.09 (s, 3H, CH_3_), 7.21-7.26 (m, 1H, H_Ar_), 7.37 (dd, *J* = 8.3, 2.2 Hz, 1H, H_Ar_), 7.43 (ddd, *J* = 8.7, 6.5, 1.1 Hz, 1H, H_Ar_), 7.58 (d, *J* = 8.3 Hz, 1H, H_Ar_), 7.65 (dd, *J* = 9.3, 7.6 Hz, 3H, 3xH_Ar_), 7.76 (d, *J* = 2.1 Hz, 1H, H_Ar_), 7.80-7.84 (m, 1H, H_Ar_), 8.01-8.06 (m, 2H, 2xH_Ar_). ^13^C NMR (101 MHz, CDCl_3_) δ : 44.5 (CH_3_), 118.2 (CH_Ar_), 119.4 (d, *J* = 60.8 Hz, C_q_), 119.5 (CH_Ar_) , 122.3 (C_q_), 123.7 (q, *J* = 9.2, 8.5 Hz, C_q_), 124.3 (CH_Ar_), 126.6 (2xCH_Ar_), 126.8 (C_q_), 128.0 (CH_Ar_), 128.3 (d, *J* = 5.4 Hz, CH_Ar_), 128.4 (d, *J* = 18.3 Hz, C_q_), 128.7 (2xCH_Ar_), 132.5 (CH_Ar_), 132.8 (C_q_), 133.6 (CH_Ar_), 140.4 (C_q_), 143.8 (C_q_), 149.6 (C_q_). ^19^F NMR (376 MHz, CDCl_3_) δ : -62.86 (CF_3_). HRMS (EI-MS) : m/z calcd for C_21_H_14_ClF_3_N_2_O_2_S : 451.0489 [M+H]^+^, found : 451.0486.

***3-(6-Fluoro-3-pyridyl)-2-(4-methylsulfonylphenyl)indazole (22).***

Compound **22** was obtained following general procedure D using derivative **12** as starting material. Purification was performed using Petroleum Ether/EtOAc (5/5) as eluent to furnish **22** as a pale yellow solid an 85% yield. Rf (Petroleum Ether/EtOAc, 5/5) : 0.28. Mp : 216-218 °C. IR (ATR diamond, cm^-1^) ν : 1585, 1501, 1491, 1387, 1357, 1311, 1300, 1257, 1145, 1093, 953, 925, 835, 776, 752, 744, 687. ^1^H NMR (250 MHz, CDCl_3_) δ : 3.11 (s, 3H, CH_3_), 7.05 (dd, *J* = 8.5, 3.1 Hz, 1H, H_Ar_), 7.19-7.26 (m, 1H, H_Ar_), 7.39-7.47 (m, 1H, H_Ar_), 7.61-7.76 (m, 4H, 4xH_Ar_), 7.82 (d, *J* = 8.8 Hz, 1H, H_Ar_), 7.99-8.07 (m, 2H, 2xH_Ar_), 8.34 (d, *J* = 2.5 Hz, 1H, H_Ar_). ^13^C NMR (101 MHz, CDCl_3_) δ : 44.5 (CH_3_), 110.4 (d, *J* = 37.7 Hz, 2xCH_Ar_), 118.1 (CH_Ar_), 119.5 (CH_Ar_), 122.6 (C_q_), 123.7 (d, *J* = 4.8 Hz, C_q_), 124.2 (CH_Ar_), 126.5 (2xCH_Ar_), 128.1 (CH_Ar_), 128.8 (2xCH_Ar_), 131.0 (C_q_), 140.4 (C_q_), 142.0 (d, *J* = 8.2 Hz, 2xCH_Ar_), 143.8 (C_q_), 148.2 (d, *J* = 15.2 Hz, CH_Ar_), 149.6 (C_q_), 163.3 (d, *J* = 243.7 Hz, C_q_). ^19^F NMR (376 MHz, CDCl_3_) δ : -65.84 (CF). HRMS (EI-MS) : m/z calcd for C_19_H_14_FN_3_O_2_S : 368.0863 [M+H]^+^, found : 368.0862.

***3-(2,6-Difluoro-3-pyridyl)-2-(4-methylsulfonylphenyl)indazole (23).***

Compound **23** was obtained following general procedure D using derivative **12** as starting material. Purification was performed using Petroleum Ether/EtOAc (5/5) as eluent to furnish **23** as a pale yellow solid in a 24% yield. Rf (Petroleum Ether/EtOAc, 5/5) : 0.43. Mp : 84-86 °C. IR (ATR diamond, cm^-1^) ν : 1591, 1504, 1487, 1455, 1408, 1364, 1310, 1295, 1271, 1147, 1091, 996, 952, 839, 770, 748, 692. ^1^H NMR (400 MHz, CDCl_3_) δ : 3.10 (s, 3H, CH_3_), 7.02 (dd, *J* = 8.2, 2.9 Hz, 1H, H_Ar_), 7.20-7.25 (m, 1H, H_Ar_), 7.43 (dd, *J* = 8.9, 6.5 Hz, 1H, H_Ar_), 7.53 (d, *J* = 8.6 Hz, 1H, H_Ar_), 7.66 (d, *J* = 8.6 Hz, 2H, 2xH_Ar_), 7.83 (d, *J* = 8.7 Hz, 1H, H_Ar_), 7.96 (q, *J* = 8.1 Hz, 1H, H_Ar_), 8.02 (d, *J* = 8.6 Hz, 2H, 2xH_Ar_). ^13^C NMR (101 MHz, CDCl_3_) δ : 44.6 (CH_3_), 107.5 (dd, *J* = 34.7, 6.1 Hz, CH_Ar_), 109.6 (dd, *J* = 28.4, 6.1 Hz, C_q_), 118.4 (CH_Ar_), 119.5 (CH_Ar_), 123.4 (C_q_), 124.4 (CH_Ar_), 125.8 (2xCH_Ar_), 126.7 (d, *J* = 4.3 Hz, C_q_), 128.1 (CH_Ar_), 129.0 (2xCH_Ar_), 140.6 (C_q_), 144.2 (C_q_), 146.3 (dd, *J* = 8.0, 3.2 Hz, CH_Ar_), 149.7 (C_q_), 159.3 (C_q_), 162.0 (dd, *J* = 252.1, 14.0 Hz, C_q_). ^19^F NMR (376 MHz, CDCl_3_) δ : -64.94 (d, *J* = 10.3 Hz, CF), -64.72 (d, *J* = 10.3 Hz, CF). HRMS (EI-MS) : m/z calcd for C_19_H_13_F_2_N_3_O_2_S : 386.0769 [M+H]^+^, found : 386.0770.

***3-(6-Methoxy-3-pyridyl)-2-(4-methylsulfonylphenyl)indazole (24).***

Compound **24** was obtained following general procedure D using derivative **12** as starting material. Purification was performed using Petroleum Ether/EtOAc (5/5) as eluent to furnish **24** as a pale yellow solid in an 85% yield. Rf (Petroleum Ether/EtOAc, 5/5) : 0.34. Mp : 146-148 °C. IR (ATR diamond, cm^-1^) ν : 1593, 1493, 1383, 1312, 1296, 1285, 1151, 1095, 1026, 1013, 956, 836, 772, 760. ^1^H NMR (400 MHz, CDCl_3_) δ : 3.10 (s, 3H, CH_3_), 4.00 (s, 3H, CH_3_), 6.82 (d, *J* = 8.5 Hz, 1H, H_Ar_), 7.16-7.20 (m, 1H, H_Ar_), 7.40 (ddd, *J* = 8.7, 6.6, 1.1 Hz, 1H, H_Ar_), 7.47 (dd, *J* = 8.4, 2.5 Hz, 1H, H_Ar_), 7.65 (d, *J* = 8.6 Hz, 1H, H_Ar_), 7.68-7.73 (m, 2H, 2xH_Ar_), 7.79 (d, *J* = 8.8 Hz, 1H, H_Ar_), 7.98-8.03 (m, 2H, 2xH_Ar_), 8.27 (d, *J* = 2.4 Hz, 1H, H_Ar_). ^13^C NMR (101 MHz, CDCl_3_) δ : 44.5 (CH_3_), 53.8 (CH_3_), 111.6 (CH_Ar_), 117.9 (CH_Ar_), 118.6 (C_q_), 120.1 (CH_Ar_), 122.4 (C_q_), 123.5 (CH_Ar_), 126.5 (2xCH_Ar_), 127.9 (CH_Ar_), 128.6 (2xCH_Ar_), 139.4 (CH_Ar_), 140.0 (C_q_), 143.5 (C_q_), 144.2 (C_q_), 147.62 (CH_Ar_), 149.6 (C_q_), 164.2 (C_q_). HRMS (EI-MS) : m/z calcd for C_20_H_17_N_3_O_2_S : 380.1063 [M+H]^+^, found : 380.1063.

***3-(5-Methoxy-3-pyridyl)-2-(4-methylsulfonylphenyl)indazole (25).***

Compound **25** was obtained following general procedure D using derivative **12** as starting material. Purification was performed using Petroleum Ether/EtOAc (5/5) as eluent to furnish **25** as a white solid in a 46% yield. Rf (Petroleum Ether/EtOAc, 5/5) : 0.14. Mp : 215-217 °C.IR (ATR diamond, cm^-1^) ν : 1594, 1508, 1454, 1415, 1331, 1309, 1291, 1267, 1233, 1179, 1144, 967, 879, 867, 852, 778, 768, 758, 711. ^1^H NMR (250 MHz, CDCl_3_) δ : 3.09 (s, 3H, CH_3_), 3.83 (s, 3H, CH_3_), 7.15 (dd, *J* = 2.8, 1.8 Hz, 1H, H_Ar_), 7.21 (ddd, *J* = 8.6, 6.6, 0.9 Hz, 1H, H_Ar_), 7.42 (ddd, *J* = 8.9, 6.6, 1.1 Hz, 1H, H_Ar_), 7.65-7.72 (m, 3H, 3xH_Ar_), 7.81 (dt, *J* = 8.8, 0.9 Hz, 1H, H_Ar_), 7.97-8.04 (m, 2H, 2xH_Ar_), 8.24 (d, *J* = 1.8 Hz, 1H, H_Ar_), 8.37 (d, *J* = 2.8 Hz, 1H, H_Ar_). ^13^C NMR (63 MHz, CDCl_3_) δ : 44.7 (CH_3_), 55.9 (CH_3_), 118.2 (CH_Ar_), 120.0 (CH_Ar_), 121.3 (CH_Ar_), 122.8 (C_q_), 124.1 (CH_Ar_), 126.2 (C_q_), 126.6 (2xCH_Ar_), 128.1 (CH_Ar_), 128.8 (2xCH_Ar_), 132.1 (C_q_), 137.7 (CH_Ar_), 140.4 (C_q_), 142.3 (CH_Ar_), 144.2 (C_q_), 149.8 (C_q_), 155.7 (C_q_). HRMS (EI-MS) : m/z calcd for C_20_H_17_N_3_O_2_S : 380.1063 [M+H]^+^, found : 380.1067.

***3-(6-Ethoxy-3-pyridyl)-2-(4-methylsulfonylphenyl)indazole (26).***

Compound **26** was obtained following general procedure D using derivative **12** as starting material. Purification was performed using Petroleum Ether/EtOAc (5/5) as eluent to furnish **26** as a white solid in a 94% yield. Rf (Petroleum Ether/EtOAc, 5/5) : 0.37. Mp : 147-149 °C. IR (ATR diamond, cm^-1^) ν : 2977, 2923, 1590, 1492, 1380, 1286, 1253, 1144, 1094, 1038, 953, 923, 837, 778, 754. ^1^H NMR (400 MHz, CDCl_3_) δ : 1.44 (t, *J* = 7.1 Hz, 3H, CH_3_), 3.09 (s, 3H, CH_3_), 4.42 (q, *J* = 7.1 Hz, 2H, CH_2_), 6.79 (dd, *J* = 8.6, 0.8 Hz, 1H, H_Ar_), 7.17 (ddd, *J* = 8.6, 6.6, 0.9 Hz, 1H, H_Ar_), 7.40 (ddd, *J* = 8.8, 6.5, 1.1 Hz, 1H, H_Ar_), 7.46 (dd, *J* = 8.6, 2.5 Hz, 1H, H_Ar_), 7.65 (dt, *J* = 8.6, 1.0 Hz, 1H, H_Ar_), 7.68-7.72 (m, 2H, 2xH_Ar_), 7.78 (dt, *J* = 8.8, 0.9 Hz, 1H, H_Ar_), 7.98-8.02 (m, 2H, 2xH_Ar_), 8.24 (dd, *J* = 2.6, 0.8 Hz, 1H, H_Ar_). ^13^C NMR (101 MHz, CDCl_3_) δ : 15.0 (CH_3_), 45.0 (CH_3_), 62.7 (CH_2_), 112.2 (CH_Ar_), 118.4 (CH_Ar_), 118.8 (C_q_), 120.6 (CH_Ar_), 122.8 (C_q_), 123.9 (CH_Ar_), 126.9 (2xCH_Ar_), 128.3 (CH_Ar_), 129.1 (2xCH_Ar_), 133.3 (C_q_), 139.8 (CH_Ar_), 140.4 (C_q_), 144.7 (C_q_), 148.0 (CH_Ar_), 150.0 (C_q_), 164.4 (C_q_). HRMS (EI-MS) : m/z calcd for C_21_H_19_N_3_O_3_S : 394.1220 [M+H]^+^, found : 394.1220.

***3-(2-Ethoxypyrimidin-5-yl)-2-(4-methylsulfonylphenyl)indazole (27).***

Compound **27** was obtained following general procedure D using derivative **12** as starting material. Purification was performed using Petroleum Ether/EtOAc (5/5) as eluent to furnish **27** as a white solid in an 85% yield. Rf (Petroleum Ether/EtOAc, 5/5) : 0.27. Mp : 217-219 °C. IR (ATR diamond, cm^-1^) ν : 2974, 1592, 1530, 1485, 1449, 1420, 1381, 1327, 1310, 1299, 1288, 1146, 1096, 1036, 954, 843, 801, 776, 754. ^1^H NMR (400 MHz, CDCl_3_) δ : 1.49 (t, *J* = 7.1 Hz, 3H, CH_3_), 3.11 (s, 3H, CH_3_), 4.50 (q, *J* = 7.1 Hz, 2H, CH_2_), 7.23 (ddd, *J* = 8.7, 6.7, 0.9 Hz, 1H, H_Ar_), 7.43 (ddd, *J* = 8.8, 6.6, 1.1 Hz, 1H, H_Ar_), 7.63 (dt, *J* = 8.7, 1.0 Hz, 1H, H_Ar_), 7.67-7.73 (m, 2H, 2xH_Ar_), 7.81 (dt, *J* = 8.9, 0.9 Hz, 1H, H_Ar_), 8.02-8.08 (m, 2H, 2xH_Ar_), 8.52 (s, 2H, 2xH_Ar_). ^13^C NMR (101 MHz, CDCl_3_) δ : 14.4 (CH_3_), 44.5 (CH_3_), 64.3 (CH_2_), 77.3 (C_q_), 117.7 (C_q_), 118.2 (CH_Ar_), 119.4 (CH_Ar_), 122.6 (C_q_), 124.2 (CH_Ar_), 126.5 (2xCH_Ar_), 128.1 (CH_Ar_), 129.0 (2xCH_Ar_), 129.2 (C_q_), 140.5 (C_q_), 143.8 (C_q_), 149.6 (C_q_), 159.3 (CH_Ar_). HRMS (EI-MS) : m/z calcd for C_20_H_18_N_4_O_3_S : 395.1172 [M+H]^+^, found : 395.1171.

***1-(3-Bromo-2-pyridyl)-N-(4-methylsulfanylphenyl)methanimine (28).***

Compound **28** was obtained following general procedure A using 3-bromo-2-aminopyridine and 15 min as time reaction and after washing the precipitate with a mixture of Petroleum Ether/EtOAc (9/1) and pentane as a brown solid in a 99% yield. IR (ATR diamond, cm^-1^) ν : 2972, 2918, 1595, 1487, 1422, 1093, 1062, 1012, 966, 819, 793, 748. ^1^H NMR (250 MHz, CDCl_3_) δ : 2.43 (s, 3H, CH_3_), 7.16 (dd, *J* = 8.1, 4.5 Hz, 1H, H_Ar_), 7.20-7.30 (m, 4H, 4xH_Ar_), 7.88 (dd, *J* = 8.1, 1.5 Hz, 1H, H_Ar_), 8.68 (dd, *J* = 4.5, 1.5 Hz, 1H, H_Ar_), 8.93 (s, 1H, H_Imi_). ^13^C NMR (63 MHz, CDCl_3_) δ : 15.9 (CH_3_), 115.6 (C_q_), 122.1 (2xCH_Ar_), 123.7 (C_q_), 125.9 (CH_Ar_), 127.1 (2xCH_Ar_), 130.9 (C_q_), 137.7 (C_q_), 141.4 (CH_Ar_), 148.1 (CH_Ar_), 156.4 (CH_Imi_). HRMS (EI-MS) : m/z calcd for C_13_H_11_BrN_2_S : 306.9899 [M+H]^+^, found : 306.9899.

***1-(4-Bromo-3-pyridyl)-N-(4-methylsulfanylphenyl)methanimine (29).***

Compound **29** was obtained following general procedure A using 4-bromo-3-formylpyridine hydrobromide and 15 min as time reaction and after washing the precipitate with EtOAc as a yellow solid in a 62% yield. Mp : 105-107 °C. IR (ATR diamond, cm^-1^) ν : 1611, 1580, 1563, 1543, 1485, 1456, 1444, 1426, 1397, 1358, 1242, 1191, 1093, 1063, 1008, 977, 966, 957, 872, 828, 819, 728, 698, 666. ^1^H NMR (400 MHz, CDCl_3_) δ : 2.51 (s, 3H, CH_3_), 7.21-7.34 (m, 4H, 4xH_Ar_), 8.43 (d, *J* = 5.3 Hz, 1H, H_Ar_), 8.81 (s, 1H, H_Ar_), 9.31 (s, 1H, H_Imi_). ^13^C NMR (101 MHz, CDCl_3_) δ : 16.2 (CH_3_), 121.9 (2xCH_Ar_), 127.5 (2xCH_Ar_), 128.1 (CH_Ar_), 130.7 (C_q_), 135.4 (C_q_), 137.7 (C_q_), 148.2 (C_q_), 150.3 (CH_Ar_), 151.6 (CH_Ar_), 155.7 (CH_Imi_). HRMS (EI-MS) : m/z calcd for C_13_H_11_BrN_2_S : 306.9899 [M+H]^+^, found : 306.9898.

***1-(3-Bromo-4-pyridyl)-N-(4-methylsulfanylphenyl)methanimine (30).***

Compound **30** was obtained following general procedure A using 3-bromo-4-formylpyridine and 15 min as time reaction and after washing the precipitate with EtOAc as a yellow solid in a 99 %yield. Mp : 79-81 °C. IR (ATR diamond, cm^-1^) ν : 2918, 1574, 1485, 1396, 1349, 1284, 1202, 1078, 1016, 953, 834, 810, 676. ^1^H NMR (400 MHz, CDCl_3_) δ : 2.52 (s, 3H, CH_3_), 7.25-7.34 (m, 4H, 4xH_Ar_), 8.05 (d, *J* = 5.0 Hz, 1H, H_Ar_), 8.60 (d, *J* = 5.0 Hz, 1H, H_Ar_), 8.81 (s, 2H, H_Ar_ et H_Imi_). ^13^C NMR (101 MHz, CDCl_3_) δ : 16.0 (CH3), 122.1 (2xCH_Ar_), 122.1 (CH_Ar_), 122.9 (C_q_), 127.3 (2xCH_Ar_), 138.5 (C_q_), 141.3 (C_q_), 147.6 (C_q_), 148.7 (CH_Ar_), 153.1 (CH_Ar_), 155.7 (CH_Imi_). HRMS (EI-MS) : m/z calcd for C_13_H_11_BrN_2_S : 306.9899 [M+H]^+^, found : 306.9901.

***1-(2-Bromo-3-pyridyl)-N-(4-methylsulfanylphenyl)methanimine (31).***

Compound **31** was obtained following general procedure A using 3-bromo-4-formylpyridine and 15 min as time reaction and after washing the precipitate with a mixture of Petroleum Ether/EtOAc mixture (9/1) as a yellow solid in a 99 %yield. Mp : 72-74 °C. IR (ATR diamond, cm^-1^) ν : 2911, 1610, 1577, 1551, 1487, 1394, 1345, 1272, 1222, 1095, 1057, 810, 797, 732. ^1^H NMR (250 MHz, CDCl_3_) δ : 2.52 (s, 3H, CH_3_), 7.20-7.34 (m, 4H, 4xH_Ar_), 7.38 (ddd, *J* = 7.7, 4.7, 0.8 Hz, 1H, H_Ar_), 8.44 (dd, *J* = 4.7, 2.1 Hz, 1H, H_Ar_), 8.49 (dd, *J* = 7.7, 2.1 Hz, 1H, H_Ar_), 8.77-8.82 (m, 1H, H_Imi_). ^13^C NMR (63 MHz, CDCl_3_) δ : 16.2 (CH_3_), 121.9 (2xCH_Ar_), 123.3 (CH_Ar_), 127.5 (2xCH_Ar_), 132.2 (C_q_), 137.2 (CH_Ar_), 137.7 (C_q_), 144.7 (C_q_), 148.1 (C_q_), 151.9 (CH_Ar_), 156.6 (CH_Imi_). HRMS (EI-MS) : m/z calcd for C_13_H_11_BrN_2_S : 306.9899 [M+H]^+^, found : 306.9901.

***2-(4-Methylsulfanylphenyl)pyrazolo[4,3-b]pyridine (32).***

Compound **32** was obtained following general procedure B using derivative **28** as starting material. The irradiation time was 5 min and purification was performed using Petroleum Ether/EtOAc (8/2 then 5/5) to give the title compound **32** as a brown solid in 29% yield. **Rf** (Petroleum Ether/EtOAc, 5/5) : 0.17. Mp : 134-136 °C. IR (ATR diamond, cm^-1^) ν : 3094, 1525, 1497, 1378, 1346, 1315, 1222, 1122, 1097, 1049, 949, 809, 786. ^1^H NMR (400 MHz, CDCl_3_) δ : 2.54 (s, 3H, CH_3_), 7.22-7.26 (m, 1H, H_Ar_), 7.36-7.43 (m, 2H, 2xH_Ar_), 7.81-7.87 (m, 2H, 2xH_Ar_), 8.11 (dt, *J* = 8.8, 1.2 Hz, 1H, H_Ar_), 8.61 (dd, *J* = 4.2, 1.4 Hz, 1H, H_Ar_), 8.65 (d, *J* = 0.9 Hz, 1H, H_Ar_). ^13^C NMR (101 MHz, CDCl_3_) δ : 15.9 (CH_3_), 121.4 (2xCH_Ar_), 121.5 (CH_Ar_), 122.0 (CH_Ar_), 126.1 (CH_Ar_), 127.3 (2xCH_Ar_), 137.6 (C_q_), 139.6 (C_q_), 139.7 (C_q_), 142.8 (C_q_), 149.1 (CH_Ar_). HRMS (EI-MS) : m/z calcd for C_13_H_11_N_3_S : 242.0746 [M+H]^+^, found : 242.0749.

***2-(4-Methylsulfanylphenyl)pyrazolo[3,4-c]pyridine (34).***

Compound **34** was obtained following general procedure B using derivative **30** as starting material. The irradiation time was 5 min and purification was performed using Petroleum Ether/EtOAc ( 5/5) to give the title compound **34** as a yellow solid in 45% yield. Rf (Petroleum Ether/EtOAc, 5/5) : 0.24. Mp : 155-157 °C. IR (ATR diamond, cm^-1^) ν : 2983, 1500, 1427, 1365, 1207, 1100, 1047, 959, 913, 810, 736. ^1^H NMR (400 MHz, CDCl_3_) δ : 2.56 (s, 3H, CH_3_), 7.38-7.43 (m, 2H, 2xH_Ar_), 7.55 (dd, *J* = 6.0, 1.4 Hz, 1H, H_Ar_), 7.82-7.89 (m, 2H, 2xH_Ar_), 8.20 (d, *J* = 6.0 Hz, 1H, H_Ar_), 8.41 (d, *J* = 0.9 Hz, 1H, H_Ar_), 9.35 (d, *J* = 1.4 Hz, 1H, H_Ar_). ^13^C NMR (101 MHz, CDCl_3_) δ : 15.8 (CH_3_), 113.5 (CH_Ar_), 120.1 (CH_Ar_), 121.8 (2xCH_Ar_), 124.6 (C_q_), 127.2 (2xCH_Ar_), 137.3 (C_q_), 138.6 (CH_Ar_), 140.3 (C_q_), 145.1 (CH_Ar_), 146.3 (C_q_). HRMS (EI-MS) : m/z calcd for C_13_H_11_N_3_S : 242.0746 [M+H]^+^, found : 242.0748.

***2-(4-Methylsulfanylphenyl)pyrazolo[3,4-b]pyridine (35).***

Compound **34** was obtained following general procedure B using derivative **30** as starting material. The irradiation time was 5 min and purification was performed using Petroleum Ether/EtOAc (5/5) to give the title compound **35** as a brown solid in 23% yield. Rf (Petroleum Ether/EtOAc, 5/5) : 0.28. Mp : 144-146 °C. IR (ATR diamond, cm^-1^) ν : 1614, 1511, 1497, 1349, 1337, 1318, 1209, 1116, 1096, 1049, 949, 812, 769, 737. ^1^H NMR (400 MHz, CDCl_3_) δ : 2.54 (s, 3H, CH_3_), 7.08 (dd, *J* = 8.4, 4.1 Hz, 1H, H_Ar_), 7.36-7.42 (m, 2H, 2xH_Ar_), 7.87-7.94 (m, 2H, 2xH_Ar_), 8.08 (dd, *J* = 8.4, 1.7 Hz, 1H, H_Ar_), 8.40 (s, 1H, H_Ar_), 8.73 (dd, *J* = 4.1, 1.7 Hz, 1H, H_Ar_). ^13^C NMR (101 MHz, CDCl_3_) δ : 15.9 (CH_3_), 115.2 (C_q_), 118.6 (CH_Ar_), 119.7 (CH_Ar_), 121.3 (2xCH_Ar_), 127.3 (2xCH_Ar_), 129.8 (CH_Ar_), 137.5 (C_q_), 139.5 (C_q_), 152.6 (CH_Ar_), 159.0 (C_q_). HRMS (EI-MS) : m/z calcd for C_13_H_11_N_3_S : 242.0746 [M+H]^+^, found : 242.0751.

***2-(4-Methylsulfonylphenyl)pyrazolo[4,3-b]pyridine (36).***

Derivative **36** was obtained following the oxone procedure described for compound **7** starting from **32** with a reaction time of 18 h. After flash chromatography with a gradient of Petroleum Ether/EtOAc (5/5 then 1/9), the title derivative was obtained as a yellow solid in a 66% yield. Rf (Petroleum Ether/EtOAc, 9/1) : 0.18. Mp : 237-239 °C. IR (ATR diamond, cm^-1^) ν : 1594, 1530, 1511, 1403, 1280, 1231, 1143, 1088, 943, 830, 808, 792, 773, 720. ^1^H NMR (400 MHz, DMSO-*d*_6_) δ : 3.32 (s, 3H, CH_3_), 7.38 (dd, *J* = 8.8, 4.0 Hz, 1H, H_Ar_), 8.11-8.19 (m, 2H, 2xH_Ar_), 8.19-8.26 (m, 1H, H_Ar_), 8.42-8.48 (m, 2H, 2xH_Ar_), 8.63 (dd, *J* = 4.0, 1.5 Hz, 1H, H_Ar_), 9.62 (d, *J* = 1.1 Hz, 1H, H_Ar_). ^13^C NMR (101 MHz, DMSO-*d*_6_) δ : 43.4 (CH_3_), 120.8 (2xCH_Ar_), 122.9 (CH_Ar_), 123.5 (CH_Ar_), 125.8 (CH_Ar_), 128.8 (2xCH_Ar_), 138.9 (C_q_), 140.0 (C_q_), 142.5 (C_q_), 143.1 (C_q_), 149.5 (CH_Ar_). HRMS (EI-MS) : m/z calcd for C_13_H_11_N_3_O_2_S : 274.0645 [M+H]^+^, found : 274.0646.

***2-(4-Methylsulfonylphenyl)pyrazolo[3,4-c]pyridine (37).***

Derivative **37** was obtained following the oxone procedure described for compound **7** starting from **34** with a reaction time of 18 h. After flash chromatography with a gradient of Petroleum Ether/EtOAc (5/5 then 2/8), the title derivative was obtained as a yellow solid in a 24% yield. Rf (Petroleum Ether/EtOAc, 5/5) : 0.11. Mp : 244-246 °C. IR (ATR diamond, cm^-1^) ν : 1594, 1506, 1422, 1357, 1291, 1210, 1140, 1088, 1051, 978, 957, 844, 807, 774, 728. ^1^H NMR (400 MHz, CDCl_3_) δ : 3.13 (s, 3H, CH_3_), 7.57 (d, *J* = 6.0 Hz, 1H, H_Ar_), 8.15-8.26 (m, 5H), 8.56 (s, 1H, H_Ar_), 9.39 (s, 1H, H_Ar_). ^13^C NMR (101 MHz, CDCl_3_) δ : 44.7 (CH_3_), 113.6 (CH_Ar_), 120.7 (CH_Ar_), 121.9 (2xCH_Ar_), 124.8 (C_q_), 129.5 (2xCH_Ar_), 139.1 (CH_Ar_), 140.70 (C_q_), 143.9 (C_q_), 145.7 (CH_Ar_), 146.8 (C_q_). HRMS (EI-MS) : m/z calcd for C_13_H_11_N_3_O_2_S : 274.0645 [M+H]^+^, found : 247.0646.

***2-(4-Methylsulfonylphenyl)pyrazolo[3,4-b]pyridine (38).***

Derivative **38** was obtained following the oxone procedure described for compound **7** starting from **35** with a reaction time of 18 h. After flash chromatography with a mixture of Petroleum Ether/EtOAc (1/9), the title derivative was obtained as a white solid in a 58% yield. Rf (Petroleum Ether/EtOAc, 1/9) : 0.23. Mp : > 250 °C. IR (ATR diamond, cm^-1^) ν : 1615, 1587, 1516, 1417, 1293, 1142, 1114, 1087, 962, 846, 841, 813, 766, 723. ^1^H NMR (400 MHz, DMSO-*d*_6_) δ : 3.33 (s, 3H, CH_3_), 7.21 (dd, *J* = 8.4, 4.1 Hz, 1H, H_Ar_), 8.18 (d, *J* = 8.4 Hz, 2H, 2xH_Ar_), 8.32 (dd, *J* = 8.4, 1.8 Hz, 1H, H_Ar_), 8.43 (d, *J* = 8.5 Hz, 2H, 2xH_Ar_), 8.74 (dd, *J* = 4.1, 1.8 Hz, 1H, H_Ar_), 9.35 (s, 1H, H_Ar_). ^13^C NMR (101 MHz, DMSO-*d*_6_) δ : 43.4 (CH_3_), 114.9 (C_q_), 118.9 (CH_Ar_), 120.9 (2xCH_Ar_), 123.0 (CH_Ar_), 128.8 (2xCH_Ar_), 130.9 (CH_Ar_), 140.0 (C_q_), 143.0 (C_q_), 153.3 (CH_Ar_), 158.5 (C_q_). HRMS (EI-MS) : m/z calcd for C_13_H_11_N_3_O_2_S : 274.0645 [M+H]^+^, found : 247.0644.

***3-(4-Fluorophenyl)-2-(4-methylsulfonylphenyl)pyrazolo[4,3-b]pyridine (39).***

A microwave vial (2-5 mL) with a stir bar was charged with a mixture of **36** (0.050 g, 0.18 mmol, 1.0 equiv), 1-Fluoro-4-iodobenzene (0.045 g, 0.20 mmol, 1.2 equiv), Ag_2_CO_3_ (0.051 g, 0.18 mmol, 1.0 equiv), PPh_3_ (0.005 g, 0.02 mmol, 0.1 equiv), Pd(dppf)Cl_2_.CH_2_Cl_2_ (0.07 g, 0.01 mmol, 0.05 equiv). The mixture was stirred and then distilled water (3 mL) was added. The vial was sealed and then put in an oil bath pre-heated at 50 °C for 18 h. After cooling, CH_2_Cl_2_ (3 mL) was added, the heterogeneous solution was stirred 10 min and then the aqueous layer was extracted twice with CH_2_Cl_2_. Organic layers were combined, dried over MgSO_4_, concentrated under vacuum and purified by flash chromatography with Petroleum Ether/EtOAc (5/5 then 2/8) as eluent to give **39** (0.050 g, 63%) as a brown solid. Rf (Petroleum Ether/EtOAc, 5/5) : 0.17. Mp : 219-221 °C. IR (ATR diamond, cm^-1^) ν : 1590, 1508, 1313, 1293, 1232, 1150, 1090, 969, 960, 845, 805, 770, 718. ^1^H NMR (400 MHz, CDCl_3_) δ : 3.11 (s, 3H, CH_3_), 7.11-7.22 (m, 2H, 2xH_Ar_), 7.33 (dd, *J* = 8.8, 4.0 Hz, 1H, H_Ar_), 7.49-7.56 (m, 2H, 2xH_Ar_), 7.68-7.77 (m, 2H, 2xH_Ar_), 7.99-8.07 (m, 2H, 2xH_Ar_), 8.13 (dd, *J* = 8.8, 1.5 Hz, 1H, H_Ar_), 8.68 (dd, *J* = 4.0, 1.5 Hz, 1H, H_Ar_). ^13^C NMR (101 MHz, CDCl_3_) δ : 44.6 (CH_3_), 116.5 (d, *J* = 22.0 Hz, 2xCH_Ar_), 123.1 (CH_Ar_), 124.0 (d, *J* = 3.6 Hz, C_q_), 126.2 (CH_Ar_), 126.7 (2xCH_Ar_), 128.8 (2xCH_Ar_), 132.0 (d, *J* = 8.3 Hz, 2xCH_Ar_), 135.5 (C_q_), 137.6 (C_q_), 140.6 (C_q_), 142.9 (C_q_), 144.4 (C_q_), 149.9 (CH_Ar_), 163.2 (d, *J* = 250.9 Hz, C_q_). ^19^F NMR (376 MHz, CDCl_3_) δ : -110.74 (CF). HRMS (EI-MS) : m/z calcd for C_19_H_14_FN_3_O_2_S : 368.0863 [M+H]^+^, found : 368.0867.

***3-(4-Fluorophenyl)-2-(4-methylsulfonylphenyl)pyrazolo[3,4-c]pyridine (40).***

Derivative **40** was obtained following the procedure described for compound **39** starting from **37**, after flash chromatography with a mixture of Petroleum Ether/EtOAc (5/5 then 2/8), as a white solid in a 75% yield. Rf (Petroleum Ether/EtOAc, 2/8) : 0.14. Mp : 195-197 °C. IR (ATR diamond, cm^-1^) ν : 1594, 1520, 1397, 1353, 1315, 1297, 1227, 1154, 1093, 969, 849, 819, 777, 722. ^1^H NMR (250 MHz, CDCl_3_) δ : 3.11 (s, 3H, CH_3_), 7.14-7.24 (m, 2H, 2xH_Ar_), 7.29-7.38 (m, 2H, 2xH_Ar_), 7.51 (dd, *J* = 6.1, 1.4 Hz, 1H, H_Ar_), 7.65-7.74 (m, 2H, 2xH_Ar_), 7.98-8.08 (m, 2H, 2xH_Ar_), 8.26 (d, *J* = 6.0 Hz, 1H, H_Ar_), 9.38 (s, 1H, H_Ar_). ^13^C NMR (101 MHz, CDCl_3_) δ : 44.6 (CH_3_), 113.4 (CH_Ar_), 116.9 (d, *J* = 22.0 Hz, 2xCH_Ar_), 124.0 (C_q_), 124.5 (d, *J* = 3.6 Hz, C_q_), 126.8 (2xCH_Ar_), 128.8 (2xCH_Ar_), 131.5 (d, *J* = 8.4 Hz, 2xCH_Ar_), 135.2 (C_q_), 139.4 (CH_Ar_), 141.0 (C_q_), 143.9 (C_q_), 145.4 (CH_Ar_), 146.1 (C_q_), 163.35 (d, *J* = 251.9 Hz, C_q_). ^19^F NMR (376 MHz, CDCl_3_) δ : -109.97 (CF). HRMS (EI-MS) : m/z calcd for C_19_H_14_FN_3_O_2_S : 368.0863 [M+H]^+^, found : 368.0862.

***3-(4-Fluorophenyl)-2-(4-methylsulfonylphenyl)pyrazolo[3,4-b]pyridine (41).***

Derivative **41** was obtained following the procedure described for compound **39** starting from **38**, after flash chromatography with a mixture of Petroleum Ether/EtOAc (5/5 then 2/8), as a pale yellow solid in a 79% yield. Rf (Petroleum Ether/EtOAc, 2/8) : 0.14. Mp : 113-115 °C. IR (ATR diamond, cm^-1^) ν : 1590, 1546, 1506, 1315, 1297, 1266, 1152, 1090, 961, 849, 780, 769, 729. ^1^H NMR (400 MHz, CDCl_3_) δ : 3.10 (s, 3H, CH_3_), 7.09-7.23 (m, 3H, 3xH_Ar_), 7.34 (q, *J* = 5.7, 4.8 Hz, 2H, 2xH_Ar_), 7.72 (d, *J* = 8.3 Hz, 2H, 2xH_Ar_), 8.01 (t, *J* = 9.4 Hz, 3H, 3xH_Ar_), 8.77-8.89 (m, 1H, H_Ar_). ^13^C NMR (101 MHz, CDCl_3_) δ : 44.4 (CH_3_), 114.7 (C_q_), 116.8 (d, *J* = 22.0 Hz, 2xCH_Ar_), 119.2 (CH_Ar_), 124.6 (d, *J* = 4.3 Hz, C_q_), 126.5 (2xCH_Ar_), 128.5 (2xCH_Ar_), 129.9 (CH_Ar_), 131.4 (d, *J* = 8.5 Hz, 2xCH_Ar_), 135.0 (C_q_), 140.3 (C_q_), 143.8 (C_q_), 144.7 (d, *J* = 6.3 Hz), 153.7 (CH_Ar_), 173.6 (d, *J* = 446.1 Hz, C_q_). ^19^F NMR (376 MHz, CDCl_3_) δ : -109.94 (CF). HRMS (EI-MS) : m/z calcd for C_19_H_14_FN_3_O_2_S : 368.0863 [M+H]^+^, found : 368.0862.

***4-[3-(4-Nitrophenyl)indazol-2-yl]benzenesulfonamide (42).***

Compound **42** was obtained following general procedure D using derivative **13** as starting material. Purification was performed using Petroleum Ether/EtOAc (5/5) as eluent to furnish **42** as a pale yellow solid in a 53% yield. **Rf** (Petroleum Ether/EtOAc, 5/5) : 0.34. Mp : 221 - 223 °C. IR (ATR diamond, cm^-1^) ν : 3265, 3154, 3046, 2359, 1594, 1516, 1400, 1339, 1303, 1286, 1162, 1097, 1017, 980, 906, 852, 939, 755, 739, 720, 696. ^1^H NMR (400 MHz, DMSO-*d*_6_) δ : 7.27 (t, *J* = 7.6 Hz, 1H, H_Ar_), 7.39 (s, 2H, NH_2_), 7.43 – 7.48 (m, 1H, H_Ar_), 7.70 (d, *J* = 8.2 Hz, 4H, 4xH_Ar_), 7.79 (dd, *J* = 27.3, 8.7 Hz, 2H, 2xH_Ar_), 7.92 (d, *J* = 8.2 Hz, 2H, 2xH_Ar_), 8.32 (d, *J* = 8.4 Hz, 2H, 2xH_Ar_). ^13^C NMR (101 MHz, DMSO-*d*_6_) δ : 117.7 (CH_Ar_), 119.9 (CH_Ar_), 121.8 (C_q_), 123.8 (CH_Ar_), 124.1 (2xCH_Ar_), 126.5 (2xCH_Ar_), 126.8 (2xCH_Ar_), 127.6 (CH_Ar_), 130.8 (2xCH_Ar_), 133.1 (C_q_), 135.3 (C_q_), 141.7 (C_q_), 144.0 (C_q_), 147.0 (C_q_), 148.6 (C_q_). HRMS (EI-MS) : m/z calcd for C_19_H_14_N_4_O_4_S : 395.0809 [M+H]^+^, found : 395.0806.

***4-[3-(4-Trimethylsilylphenyl)indazol-2-yl]benzenesulfonamide (43).***

Compound **43** was obtained following general procedure D using derivative **13** as starting material. Purification was performed using Petroleum Ether/EtOAc (54/45) as eluent to furnish **42** as a beige solid in a 69% yield. Rf (Petroleum Ether/EtOAc, 5/5) : 0.47. Mp : 220 - 222 °C. IR (ATR diamond, cm^-1^) ν : 3262, 3160, 3043, 2948, 2356, 1594, 1505, 1394, 1360, 1339, 1305, 1248, 1162, 1095, 1020, 977, 906, 835, 754, 734, 725, 688. ^1^H NMR (250 MHz, DMSO-*d*_6_) δ : 0.28 (s, 9H), 7.15 – 7.21 (m, 1H, H_Ar_), 7.37 – 7.44 (m, 3H, 3xH_Ar_), 7.53 (s, 2H, NH_2_), 7.62 – 7.79 (m, 6H), 7.88 – 7.92 (m, 2H, 2xH_Ar_). ^13^C NMR (63 MHz, DMSO-*d*_6_) δ : -1.2 (3xCH_3_), 117.4 (CH_Ar_), 120.4 (CH_Ar_), 121.4 (C_q_), 122.9 (CH_Ar_), 126.4 (2xCH_Ar_), 126.6 (2xCH_Ar_), 127.3 (CH_Ar_), 128.6 (2xCH_Ar_), 129.2 (C_q_), 133.8 (2xCH_Ar_), 135.2 (C_q_), 140.6 (C_q_), 142.2 (C_q_), 148.5 (C_q_). HRMS (EI-MS) : m/z calcd for C_22_H_23_N_3_O_2_SSi : 422.1353 [M+H]^+^, found : 422.1352.

***4-[3-(4-Iodophenyl)indazol-2-yl]benzenesulfonamide (44).***

To a suspension of **43** (0.500 g, 1.19 mmol) in dry CH_2_Cl_2_ (10 mL) at 0 °C, ICl 1M in CH_2_Cl_2_ (2.37 mL, 2.37 mmol, 2 .0 equiv) was added dropwise and then the mixture was stirred 1.5 h at room temperature. Et_2_O (18 mL) was added and the resulting precipitate was filtered, washed successively with a saturated aqueous Na_2_SO_4_ solution, cold water and Et_2_O. The residue was dried under vacuum to afford the desired compound **44** (0.560 g, 99 %) as a yellow solid. Mp : > 250 °C. IR (ATR diamond, cm^-1^) ν : 3317, 3217, 2880, 1634, 1587, 1512, 1501, 1406, 1343, 1294, 1253, 1169, 1099, 1058, 1022, 1008, 952, 894, 835, 821, 772, 748, 725, 712, 678. ^1^H NMR (250 MHz, DMSO-*d*_6_) δ : 7.14 – 7.28 (m, 3H, 3xH_Ar_), 7.41 (ddd, *J* = 8.8, 6.6, 1.1 Hz, 1H, H_Ar_), 7.52 (s, 2H, NH_2_), 7.62 – 7.71 (m, 3H, 3xH_Ar_), 7.76 (dt, *J* = 8.7, 0.9 Hz, 1H, H_Ar_), 7.83 – 7.94 (m, 4H, 4xH_Ar_). ^13^C NMR (63 MHz, DMSO-*d*_6_) δ : 95.7 (C_q_), 117.5 (CH_Ar_), 120.2 (CH_Ar_), 121.4 (C_q_), 123.1 (CH_Ar_), 126.4 (2xCH_Ar_), 126.7 (2xCH_Ar_), 127.4 (CH_Ar_), 128.3 (C_q_), 131.5 (2xCH_Ar_), 134.3 (C_q_), 137.9 (2xCH_Ar_), 141.9 (C_q_), 143.8 (C_q_), 148.5 (C_q_). HRMS (EI-MS) : m/z calcd for C_19_H_14_IN_3_O_2_S: 475.9924 [M+H]^+^, found : 475.9922.

***4-[3-(4-Tributylstannylphenyl)indazol-2-yl]benzenesulfonamide (45).***

A microwave vial (10-20 mL) with a stir bar was charged with a mixture of **44** (0.400 g, 0.84 mmol) and dry dioxane (20 mL). The mixture was degassed 15 min and then Hexa-n-butylditin (1.45 mL, 2.78 mmol, 3.3 equiv) and Pd(PPh_3_)_4_ (0.097 g, 0.08 mmol, 0.1 equiv) were added. The vial was sealed and then heated to 90 °C, 2 h. After cooling, the mixture was concentrated and purified by silica gel column chromatography with Petroleum Ether/EtOAc (8/2 then 5/5) as eluent to afford the desired compound **45** (0.179 g, 33 %) as a white solid. Rf (Petroleum Ether/EtOAc, 5/5) : 0.60. Mp : 207 - 209 °C. IR (ATR diamond, cm^-1^) ν : 3269, 3154, 3059, 2955, 2922, 2850, 1627, 1593, 1540, 1504, 1462, 1413, 1392, 1361, 1340, 1302, 1287, 1227, 1162, 1097, 1068, 1019, 996, 977, 904, 837, 811, 756, 740, 718. ^1^H NMR (400 MHz, DMSO-*d*_6_) δ : 0.78 – 0.92 (m, 9H, 3xCH_3_), 0.97 – 1.17 (m, 6H, 3xCH_2_), 1.30 (h, *J* = 7.3 Hz, 6H, 3xCH_2_), 1.36 – 1.71 (m, 6H, 3xCH_2_), 7.14 – 7.21 (m, 1H, H_Ar_), 7.35 (d, *J* = 7.3 Hz, 2H, 2xH_Ar_), 7.37 – 7.42 (m, 1H, H_Ar_), 7.51 (s, 2H, NH_2_), 7.56 (d, *J* = 7.5 Hz, 2H, 2xH_Ar_), 7.64 (d, *J* = 8.3 Hz, 2H, 2xH_Ar_), 7.69 (d, *J* = 8.5 Hz, 1H, H_Ar_), 7.75 (d, *J* = 8.8 Hz, 1H, H_Ar_), 7.87 (d, *J* = 8.2 Hz, 2H, 2xH_Ar_). ^13^C NMR (101 MHz, DMSO-*d*_6_) δ : 9.2 (3xCH_2_), 13.5 (3xCH_3_), 26.6 (3xCH_2_), 28.5 (3xCH_2_), 117.4 (CH_Ar_), 120.5 (CH_Ar_), 121.4 (C_q_), 122.8 (CH_Ar_), 126.2 (2xCH_Ar_), 126.5 (2xCH_Ar_), 127.3 (CH_Ar_), 128.4 (C_q_), 128.8 (2xCH_Ar_), 135.3 (C_q_), 136.7 (2xCH_Ar_), 142.20 (C_q_), 142.8 (C_q_), 143.6 (C_q_), 148.5 (C_q_). HRMS (EI-MS) : m/z calcd for C_31_H_41_N_3_O_2_SSn : 640.2019 [M+H]^+^, found : 640.2022.

***(4-Tert-butylphenyl)-[4-[2-(4-sulfamoylphenyl)indazol-3-yl]phenyl]iodonium, 4-methylbenzenesulfonate*** ***(46).***

Under inert atmosphere, to a solution of Moser reagent (0.042 g, 0.09 mmol, 1.5 equiv) in CH_3_CN (0.5 mL), CH_2_Cl_2_ (0.5 mL) and **45** (0.040 g, 0.06 mmol) were added. The mixture was stirred 18 h at room temperature and then the solvent was removed under inert gas flux. Successively, absolute EtOH (0.5 mL) and then Et_2_O (7.0 mL) was added and the resulting precipitate was filtered, washed with Et_2_O and dried under vacuum to give **46** (0.036 g, 73 %) as a white solid. Mp : 163 - 165 °C. IR (ATR diamond, cm^-1^) ν : 3202, 3063, 2961, 1594, 1503, 1462, 1395, 1362, 1333, 1305, 1160, 1120, 1106, 1032, 1008, 970, 924, 839, 815, 747, 711, 680. ^1^H NMR (400 MHz, DMSO-*d*_6_) δ : 1.28 (s, 9H, 3xCH_3_), 2.28 (s, 3H, CH_3_), 7.11 (d, *J* = 7.7 Hz, 2H, 2xH_Ar_), 7.21 (t, *J* = 7.6 Hz, 1H, H_Ar_), 7.43 (t, *J* = 7.7 Hz, 1H, H_Ar_), 7.48 (d, *J* = 7.8 Hz, 2H, 2xH_Ar_), 7.53 – 7.70 (m, 9H, NH_2_ & 7xH_Ar_), 7.79 (d, *J* = 8.6 Hz, 1H, H_Ar_), 7.88 (d, *J* = 8.3 Hz, 2H, 2xH_Ar_), 8.19 (d, *J* = 8.1 Hz, 2H, 2xH_Ar_), 8.33 (d, *J* = 8.2 Hz, 2H, 2xH_Ar_). ^13^C NMR (101 MHz, DMSO-*d*_6_) δ : 20.7 (CH_3_), 30.7 (3xCH_3_), 34.9 (C_q_), 112.6 (C_q_), 115.8 (C_q_), 117.6 (CH_Ar_), 120.0 (CH_Ar_), 121.6 (C_q_), 123.5 (CH_Ar_), 125.4 (2xCH_Ar_), 126.6 (2xCH_Ar_), 126.7 (2xCH_Ar_), 127.5 (CH_Ar_), 128.0 (2xCH_Ar_), 128.9 (2xCH_Ar_), 132.4 (2xCH_Ar_), 133.4 (C_q_), 135.1 (2xCH_Ar_), 135.7 (2xCH_Ar_), 136.7 (C_q_), 137.5 (C_q_), 141.7 (C_q_), 144.0 (C_q_), 145.8 (C_q_), 148.5 (C_q_), 155.4 (C_q_). HRMS (EI-MS) : m/z calcd for C_36_H_34_IN_3_O_5_S_2_ : 608.0863 [M]^+^, found : 608.0860.

***4-[3-[4-(4,4,5,5-Tetramethyl-1,3,2-dioxaborolan-2-yl)phenyl]indazol-2-yl]benzenesulfonamide (47).***

A microwave vial (0.5 - 2 mL) with a stir bar was charged with **15** (0.600 g, 1.70 mmol ), 1,4-benzenediboronic acid bis(pinacol) ester (1.687 g, 5.11 mmol, 3.00 equiv) and dioxane (5.6 mL). The mixture and Na_2_CO_3_ aqueous solution 2M (5.54 mL, 11.07 mmol, 6.5 equiv) were degassed 15 min with inert gas and then Pd(PPh_3_)_4_ (0.066 g, 0.06 mmol, 0.10 equiv) was added. The vial was sealed and then placed in the microwave cavity. After 40 min of irradiation at 100 °C, the mixture was poured into water (15.0 mL) and the aqueous layer was extracted twice with EtOAc (2x20 mL). Combined organic layers were dried over MgSO_4_, filtered, concentrated and purified by silica gel column chromatography with Petroleum Ether/EtOAc (5/5) as eluent to give **180** (0.423 g, 52 %) as a white solid. Rf (Petroleum Ether/EtOAc, 5/5) : 0.28. Mp : > 250 °C. IR (ATR diamond, cm^-1^) ν : 3262, 3160, 2977, 2353, 1611, 1593, 1506, 1399, 1359, 1341, 1301, 1267, 1215, 1162, 1145, 1094, 1020, 977, 962, 904, 839, 755, 738, 711, 657. ^1^H NMR (250 MHz, DMSO-*d*_6_) δ : 1.31 (s, 12H, 4xCH_3_), 7.16 – 7.24 (m, 1H, H_Ar_), 7.36 – 7.46 (m, 3H, 3xH_Ar_), 7.50 (s, 2H, 2xH_Ar_), 7.62 – 7.68 (m, 2H, 2xH_Ar_), 7.75 (dt, *J* = 7.5, 5.7 Hz, 4H, 4xH_Ar_), 7.87 – 7.93 (m, 2H, 2xH_Ar_). ^13^C NMR (63 MHz, DMSO-*d*_6_) δ : 24.6 (4xCH_3_), 83.9 (2xC_q_), 117.5 (CH_Ar_), 120.2 (CH_Ar_), 121.4 (C_q_), 123.1 (CH_Ar_), 126.3 (2xCH_Ar_), 126.6 (2xCH_Ar_), 127.2 (C_q_), 127.4 (CH_Ar_), 128.9 (2xCH_Ar_), 131.6 (C_q_), 134.8 (2xCH_Ar_), 134.9 (C_q_), 142.0 (C_q_), 143.7 (C_q_), 148.5 (C_q_). HRMS (EI-MS) : m/z calcd for C_24_H_25_BN_3_O_4_S : 476.1814 [M+H]^+^, found : 476.1812.

# Radiochemistry

^18^F production was performed on a PETTrace cyclotron (GE Healthcare) *via* the classical ^18^O(p,n)^18^F reaction at Cyclopharma laboratories. Warm radiosyntheses were performed in a hot cell using a small amount of radioactivity (50 MBq maximum) and a dry heater. Hot radiosyntheses were performed on a FXFN® module (GE Healthcare). QMA cartridges and Sep-Pak® light C-18 were provided by Waters. Microwave irradiation was realized on a PETwave® (CEM). All chemical reagents were obtained from Sigma-Aldrich, ABX and VWR. Purifications were performed on a Luna C18(2) 10x250mm 10 µm 100A (Phenomenex^®^) with ammonium acetate 0.1 M/CH_3_CN as mobile phase at a 4 mL flow rate. QC was performed on an Ultimate 3000 HPLC (Thermo) equipped with a UV detector (240 nm) and a bioscan radiodetector. The column used was a Luna C18(2) 100A 4.6 mm x 250 X 5µm (Phenomenex) at a flow rate of 1mL/min with ammonium acetate 0.1 M/ CH_3_CN as mobile phase, and the method was applied for the different radiolabeling strategy approaches.

***4-[3-(4-[^18^F]Fluorophenyl)indazol-2-yl]benzenesulfonamide [^18^F]16.***

*From the nitro precursor 42.* [^18^F]-fluoride solution (20-40 GBq) was fixed on a QMA® cartridge (preconditioned with 10 mL of 0.5 M K_2_CO_3_ and 10 mL of deionized water), and eluted with 1 mL of a solution of 3.8 mg K_2_CO_3_ (0.275 mM) in 285 µL of water and 7.2 mg of Kyptofix_2.2.2_ (0.191 mM) in 715 µL of MeCN. The solution based on complex [^18^F]-fluoride was transferred into a reactor and dried twice under azeotropic atmosphere with 1 mL of MeCN. After drying the [^18^F]-fluoride complex was ready for adding the precursor. 2 mg or the nitro precursor **42** dissolved in 1 mL of DMSO was added and transferred to the microwave device. The reaction was heated at 180 °C for 10 min. Incorporation yield was evaluated by QC HPLC on an aliquot. Due to the low yields observed (2-3 %) no HPLC purification was carried out.

*From the iodonium salt precursor 46.* The eluent solution was modified based on Neumann *et al*. [13]. In this case the eluent solution consisted of 0.9 mL of the solution of 1 mg K_2_CO_3_ in 90 µL of water and 7.0 mg of Kyptofix_2.2.2_ in 810 µL of MeCN. Azeotropic drying was performed as described above. On automation conditions, 2 mg of iodonium precursor **46** dissolved in 500 µL of DMF and 3.7 mg of Cu(OTf)_2_ dissolved in 500 µL of DMF were added to the dried fluorine. The reaction was heated 20 min at 100 °C, then cooled, diluted with water (5 mL) and crude product trapped on a *t*-C18 cartridge. Elution from the cartridge was performed by using 1 mL of CH_3_CN diluted with 1 mL of ammonium acetate 0.1 M prior to HPLC injection. Purification was done with ammonium acetate 0.1 M/CH_3_CN(45/55) as mobile phase.

*From the boronic ester.* The eluent solution was adapted from Mossine *et al*. [14] and consisted of 5 mg of KOTf, and 50 µg of K_2_CO_3_ in 550 µl of water. 1 mL of CH_3_CN was added and azeotropic drying was carried out as described above. On automation conditions, 2 mg of boronic ester precursor **47** around 3.5 mg of Cu(OTf)_2_ are dissolved in 40 µL of pyridine and 960 µL of DMF. The reaction was heated 20 min at 130 °C, then cooled, diluted with water (8 mL) and crude product was trapped on a *t*-C18 cartridge. Elution from the cartridge was performed by using 2 mL of CH_3_CN diluted with 2 mL of ammonium acetate 0.1 M prior to HPLC injection. Purification was performed using ammonium acetate 0.1 M/ CH_3_CN (50/50) as mobile phase.

For the formulation of the final compound, after HPLC purification, dilution of the collected fraction with water (30 mL) followed by trapping on a *t*-C18 cartridge and with 5 mL water was performed. The compound was eluted from the cartridge by 1 mL of injectable ethanol and formulation was completed with 9 mL of 0.9 % of NaCl.

# Biological evaluation

***In vitro binding assays.***

The inhibitory effect on COX activity was evaluated for each synthesized compound using an enzymatic fluorescence-based cyclooxygenase inhibitor screening assay kit (Cayman Chemical catalog number 700100, No. 760111, lots: 0452210, 0453645, 0455834 and 0461794, Cayman Chemical, Ann Arbor, MI, USA) according to the manufacturer’s protocol. SC-560 and DuP-697, supplied in the assay kit, were respectively used as selective inhibitors of COX-1 and COX-2 activity, and the potent and selective COX-2 inhibitor celecoxib (obtained from Sigma Aldrich, Lyon, France), was used as an additional reference compound. The COX inhibitors were assayed in concentrations ranging from 10^-8^ M to 10^-5^ M, either for COX-1 or 2 inhibition assays. MARS data analysis software (V2.41) was used to calculate the IC_50_ values. This assay measures the heme-catalyzed hydroperoxidase activity of ovine COX-1 and human recombinant COX-2 by monitoring the appearance of the fluorescent compound resorufin, produced by the reaction between a hydroperoxy-endoperoxide, which is itself the result of the peroxidase activity of COX on arachidonic acid, and 10-acetyl-3,7-dihydroxyphenoxazine^13^.

***Statistical analysis.***

All data are presented as mean ± standard deviation. Statistical methods included one-way analysis of variance (ANOVA) followed by Mann- Whitney test to determine significant difference. Statistics were performed using the XLStat software®. A p-value of 0.05 was considered a threshold criterion of statistical significance. For in vitro binding assay, values are means of triplicates, excepted for DuP-697 (n = 27) and Celecoxib (n = 5) which serves as reference in the assays.

***Ex vivo* cerebral biodistribution studies.**

*Animals.* All procedures were conducted in accordance with the requirements of the European Community Council for the care of laboratory animals 2010/63/EU, and approved by the regional ethical committee (Authorization N°2015022011523044). Experiments were carried out on adult Wistar rats (Centre d’Elevage René Janvier, Le Genest-St-Isle, France) weighing 276 ± 19 g at the beginning of the experiments. Animals were housed in groups of two per cage in a temperature and humidity controlled environment (temperature 21 °C ± 1 °C; hygrometry 55% ± 5%), under a 12 h light/dark cycle with food and water available ad libitum.

*Surgery:* Surgery was performed under isoflurane (AErrane, Baxter, France) anaesthesia (4% for anaesthesia induction and thereafter 2% for its maintenance, in N_2_O-O_2_: 70%-30%). The animal's body temperature (36.8 ± 0.5 °C) was monitored with a homoeothermic probe throughout surgery. A unilateral striatal QA lesion was induced in 18 adult male Wistar rats as previously described^14^. This procedure results in a well-delineated reproducible excitotoxic lesion to the striatum and ipsilateral cortex. Rats were placed in a stereotaxic David Kopf apparatus (tooth bar: −3.3 mm, and unilaterally injected with 150 nmol of QA (Sigma-Aldrich, Lyon, France) into the right striatum (injection rate: 0.5 𝜇L/min) using a 25-𝜇L microsyringe (Hamilton, Bonaduz, Switzerland) and a micropump (KD Scientific, Holliston, MA, USA). Two microliters of QA were injected at the following coordinates: AP: +0.7 mm; ML: -3 mm; DV: -5.5 mm from bregma, according to Paxinos and Watson^15^. The injection syringe was left in place for an additional 4 min time to avoid QA backflow and then slowly removed. The scalp was sutured and animals returned to their cages and examined daily until sacrifice.

***Ex vivo cerebral biodistribution of [^18^F]16, and blocking studies.***

*Ex vivo* cerebral biodistribution studies were performed with [^18^F]**16** in QA lesioned animals at 7 days post lesion (n=6 per group). Rats were i.v. injected (penis vein) with [^18^F]**16** (2.5 ± 1 MBq) in 300 μL normal saline). Nonspecific binding of [^18^F]**16** was investigated in 2 other groups of QA lesioned rats, by preinjection, 15 min prior to the radiotracer, of an excess (3 mg/kg) of either unlabeled compound **16**, or celecoxib. Animals were sacrificed by decapitation 60 min post [^18^F]**16** injection. The whole brain was quickly removed and several brain areas (cerebellum, left and right hippocampus, left and right frontal cortex, and left and right striatum), together with the femur, to detect potential *in vivo* defluorination of the tracer, were dissected and weighed, and their radioactivity was measured using an automated gamma counter (2480 Gamma counter Wizard, Perkin Elmer). The percentage of injected dose per gram tissue (%ID/g) was calculated by comparison with samples to standard dilutions of the initial injected solution.

*Statistical analysis:* Results were expressed as mean ± standard error of the mean (SEM). Groups of rats (control *vs* unlabeled compound **16** preinjection, or control *vs* celecoxib preinjection) were compared using the Mann–Whitney test. Comparisons of binding between ipsi- and contralateral brain structures were performed using the Wilcoxon one-tailed test. The level of significance was p<0.05 (XLStat software^®^).

***1-(2-Bromophenyl)-N-(4-fluorophenyl)methanimine (1).***

***1-(2-Bromophenyl)-N-(4-methylsulfanylphenyl)methanimine (2).***

***1-(2-Bromophenyl)-N-(4-methylsulfonylphenyl)methanimine (3).***

***4-[(2-Bromophenyl)methyleneamino]benzenesulfonamide (4).*****

**

***N'-[4-[(2-Bromophenyl)methyleneamino]phenyl]sulfonyl-N,N-dimethyl-formamidine (9).***

***2-(4-Fluorophenyl)indazole (5).***

***2-(4-Methylsulfanylphenyl)indazole (6).***

***2-(4-Methylsulfonylphenyl)indazole (7).***

***4-Indazol-2-ylbenzenesulfonamide (8).***

***3-Bromo-2-(4-fluorophenyl)indazole (11).***

***3-Bromo-2-(4-methylsulfonylphenyl)indazole (12).***

***4-(3-Bromoindazol-2-yl)benzenesulfonamide (13).***

**

**

***3-(4-Fluorophenyl)-2-(4-methylsulfonylphenyl)indazole (14).***

**

**

***2-(4-Fluorophenyl)-3-(4-methylsulfonylphenyl)indazole (15).***

***3-(4-Fluorophenyl)-2-(4-methylsulfonylphenyl)pyrazolo[4,3-b]pyridine (39).***

***3-(4-Fluorophenyl)-2-(4-methylsulfonylphenyl)pyrazolo[3,4-c]pyridine (40).***

***3-(4-Fluorophenyl)-2-(4-methylsulfonylphenyl)pyrazolo[3,4-b]pyridine (41).***

***3-(3-Fluoro-4-methyl-phenyl)-2-(4-methylsulfonylphenyl)indazole (18).***

***3-(4-Ethoxy-3-fluoro-phenyl)-2-(4-methylsulfonylphenyl)indazole (19).***

***3-[4-Ethoxy-3-(trifluoromethyl)phenyl]-2-(4-methylsulfonylphenyl)indazole (20).***

***3-[4-Chloro-3-(trifluoromethyl)phenyl]-2-(4-methylsulfonylphenyl)indazole (21).***

***3-(6-Fluoro-3-pyridyl)-2-(4-methylsulfonylphenyl)indazole (22).***

***3-(2,6-Difluoro-3-pyridyl)-2-(4-methylsulfonylphenyl)indazole (23).***

***3-(6-Methoxy-3-pyridyl)-2-(4-methylsulfonylphenyl)indazole (24).***

***3-(5-Methoxy-3-pyridyl)-2-(4-methylsulfonylphenyl)indazole (25).***

***3-(6-Ethoxy-3-pyridyl)-2-(4-methylsulfonylphenyl)indazole (26).***

***3-(2-Ethoxypyrimidin-5-yl)-2-(4-methylsulfonylphenyl)indazole (27).***

***4-[3-(4-Fluorophenyl)indazol-2-yl]benzenesulfonamide (16).***

***4-[3-(3-Fluorophenyl)indazol-2-yl]benzenesulfonamide (17).***

***1-(3-Bromo-2-pyridyl)-N-(4-methylsulfanylphenyl)methanimine (28).***

***2-(4-Methylsulfanylphenyl)pyrazolo[4,3-b]pyridine (32).***

***2-(4-Methylsulfonylphenyl)pyrazolo[4,3-b]pyridine (36).***

***1-(4-Bromo-3-pyridyl)-N-(4-methylsulfanylphenyl)methanimine (29).***

**

**

***1-(3-Bromo-4-pyridyl)-N-(4-methylsulfanylphenyl)methanimine (30).***

***2-(4-Methylsulfanylphenyl)pyrazolo[3,4-c]pyridine (34).***

***2-(4-Methylsulfonylphenyl)pyrazolo[3,4-c]pyridine (37).***

***1-(2-Bromo-3-pyridyl)-N-(4-methylsulfanylphenyl)methanimine (31).***

***2-(4-Methylsulfanylphenyl)pyrazolo[3,4-b]pyridine (35).***

***2-(4-Methylsulfonylphenyl)pyrazolo[3,4-b]pyridine (38).***

***4-[3-(4-Nitrophenyl)indazol-2-yl]benzenesulfonamide (42).***

***4-[3-(4-Trimethylsilylphenyl)indazol-2-yl]benzenesulfonamide (43).***

***4-[3-(4-Iodophenyl)indazol-2-yl]benzenesulfonamide (44).***

***4-[3-(4-Tributylstannylphenyl)indazol-2-yl]benzenesulfonamide (45).***

***(4-Tert-butylphenyl)-[4-[2-(4-sulfamoylphenyl)indazol-3-yl]phenyl]iodonium, 4-methylbenzenesulfonate*** ***(46).***

***4-[3-[4-(4,4,5,5-Tetramethyl-1,3,2-dioxaborolan-2-yl)phenyl]indazol-2-yl]benzenesulfonamide (47).***
